# A Phase 2 Study of S-588410 Maintenance Monotherapy for Platinum-Treated Advanced or Metastatic Urothelial Carcinoma

**DOI:** 10.3233/BLC-211592

**Published:** 2022-06-03

**Authors:** Nobuaki Shimizu, Syed A. Hussain, Wataru Obara, Toshinari Yamasaki, Satoru Takashima, Takahiro Hasegawa, Motofumi Iguchi, Kenji Igarashi, Osamu Ogawa, Tomoaki Fujioka

**Affiliations:** a Department of Urology, Gunma Prefectural Cancer Center, Ota, Gunma, Japan; b The Clatterbridge Cancer Centre, Wirral, UK; c Department of Urology, Iwate Medical University, Morioka, Iwate, Japan; d Department of Urology, Kyoto University Graduate School of Medicine, Kyoto, Japan; e Clinical Development, Shionogi & Co., Ltd., Osaka, Japan; f Biostatistics Center, Shionogi & Co., Ltd., Osaka, Japan; g Medical Affairs, Shionogi & Co., Ltd., Osaka, Japan; h Project Management, Shionogi & Co., Ltd., Osaka, Japan

**Keywords:** S-588410, cancer peptide vaccine, maintenance therapy, urothelial carcinoma, cytotoxic T lymphocytes

## Abstract

**BACKGROUND:**

Effective maintenance therapy for urothelial carcinoma (UC) is needed to delay progression after first-line chemotherapy.

**OBJECTIVE:**

To evaluate S-588410, a cancer peptide vaccine containing five human leukocyte antigen (HLA)-A^*^24:02-restricted epitope peptides derived from five cancer-testis antigens (DEPDC1, MPHOSPH1, URLC10, CDCA1, and KOC1) in chemotherapy-treated, clinically stable patients with advanced or metastatic UC.

**MATERIALS AND METHODS:**

This open-label, international, phase 2 trial enrolled patients with UC who had completed≥4 cycles of first-line platinum-containing chemotherapy without disease progression. Forty-five HLA-A^*^24:02-positive patients received subcutaneous injections of S-588410 (Montanide ISA 51 VG with 1 mg/mL of each peptide) weekly for 12 weeks then once every 2 weeks thereafter for up to 24 months. Thirty-six HLA-A^*^24:02-negative patients did not receive S-588410 (observation group). The primary endpoint was the rate of cytotoxic T-lymphocyte (CTL) induction against≥1 of the peptides at 12 weeks.

**RESULTS:**

The CTL induction rate in the S-588410 group was 93.3% (*p* < 0.0001, one-sided binomial test with a rate of≤50% as the null hypothesis). The antitumor response rate was 8.9% in the S-588410 group and 0% in the observation group; median progression-free survival was 18.1 versus 12.5 weeks and median overall survival was 71.0 versus 99.0 weeks, respectively. The most frequent treatment-emergent adverse event was injection-site reactions (47 events, grades 1–3) reported in 93.3% (*n* = 42/45) of participants.

**CONCLUSIONS:**

S-588410 demonstrated a high CTL induction rate, acceptable safety profile, and modest clinical response, as maintenance therapy in participants with advanced or metastatic UC who had received first-line platinum-based chemotherapy (EudraCT 2013-005274-22).

## INTRODUCTION

Bladder cancer had a reported global incidence of approximately 573,000 new cases and 213,000 deaths annually according to global cancer statistics in 2020 [1]. Bladder cancer is more common in men, for whom it ranks as the sixth most common cancer and ninth leading cause of cancer deaths, with respective incidence and mortality rates of 9.5 and 3.3 per 1,000 globally, about four times those of women [1]. Urothelial carcinoma is the most common type of bladder cancer.

Cisplatin-based combination chemotherapy is a widely used and well-established first-line chemotherapy regimen for the treatment of bladder cancer [2]. In patients with locally advanced or metastatic disease, although the initial response rate from gemcitabine and cisplatin (GC) and methotrexate, vinblastine, doxorubicin, and cisplatin (MVAC) therapy can be as high as 50%, survival rates remain low, with a median progression-free survival (PFS) of approximately 8 months and median overall survival (OS) of approximately 15 months [3–5].

Owing to the limited therapeutic effect and the difficulty of repeating cisplatin-based regimens for more than six cycles, there is a need for an effective maintenance therapy to prolong time to progression after completing first-line chemotherapy. This need is reinforced by the lack of approved second-line chemotherapeutics for advanced and metastatic bladder cancer in the US and Japan, although vinflunine monotherapy has been approved in Europe [6, 7]. In the maintenance setting, clinical studies have shown that vinflunine may be associated with OS and PFS benefits compared with best supportive care, but the results are not conclusive [8, 9]. Other drugs such as sunitinib and lapatinib, have been investigated as maintenance therapy in advanced bladder cancer with little success [10, 11].

Recently, immune checkpoint inhibitors have been approved as first-line and second-line treatment for advanced or metastatic urothelial cancer patients who are not eligible for cisplatin-containing chemotherapy in the US and Europe based on the KEYNOTE (pembrolizumab) or IMvigor (atezolizumab) studies [12–17]. The efficacy of immune checkpoint inhibitors appears to be associated with the existence of tumor-infiltrating lymphocytes (TILs) and the expression of programmed death ligand 1 (PD-L1) in tumors [18]. Because cancer vaccines increase antigen-specific cytotoxic T lymphocytes (CTLs) in the circulation and promote the recruitment of CTLs at the tumor site as TILs, they might be efficacious in urothelial cancer. S-588410 is a cancer peptide vaccine comprised of five human leukocyte antigen (HLA)-A^*^24:02-restricted epitope peptides derived from five cancer-testis antigens: DEP domain-containing 1 (DEPDC1), M-phase phosphoprotein 1 (MPHOSPH1), up-regulated lung cancer 10 (URLC10), cell division cycle-associated protein 1 (CDCA1), and KH domain-containing protein overexpressed in cancer 1 (KOC1). High expression of these antigens was observed in bladder cancer and is associated with tumor growth [19–23]. A cancer peptide vaccine derived from DEPDC1 and MPHOSPH1 has demonstrated CTL induction, efficacy, and an acceptable safety profile in bladder cancer [24–26]. The inclusion of five different tumor-associated peptides in S-588410 was intended to induce expansion of multiple CTLs with different antigen specificities, thereby circumventing the ability of the tumor to evade a cytotoxic T-cell response. In a previous study, S-588410 was well tolerated by patients with esophageal cancer and increased CTL, TIL, and PD-L1 expression in tumors [27].

With a more acceptable safety profile expected with a cancer peptide vaccine than chemotherapy, S-588410 may fill the yet unmet medical need of maintenance therapy for patients with advanced or metastatic bladder cancer, whose disease responds or remains stable after completion of first-line chemotherapy. This open-label phase 2 study aimed to evaluate the immune response, safety, and efficacy of S-588410 as maintenance monotherapy after response to first-line platinum-containing chemotherapy in HLA-A^*^24:02-positive patients with advanced or metastatic urothelial carcinoma.

## MATERIALS AND METHODS

### Study design, treatment, and ethics

The study was an open-label, multicenter study consisting of two study periods (Supplemental Fig. S1). Eligible participants were enrolled and assigned to the S-588410 group or an observation group (no treatment) depending on their HLA-A genotype, which was determined by polymerase chain reaction analysis. HLA-A^*^24:02-positive participants were treated with S-588410 (S-588410 group), whereas HLA-A^*^24:02-negative participants were not treated (observation group). The treatment or observation periods were 24 months (104 weeks) after enrollment. Participants in the S-588410 group were treated with 1 mL of S-588410 emulsion containing 1 mg of each of five antigen-derived peptides (DEPDC1, MPHOSPH1, URLC10, CDCA1, and KOC1) in Montanide ISA 51 VG (Seppic S.A., Paris, France), subcutaneously in the inguinal, axillary, or cervical region once weekly for 12 weeks and once every 2 weeks thereafter for up to 24 months.

This study (clinical trial number: EudraCT 2013-005274-22) was conducted at 62 centers in Japan, the UK, France, and Bulgaria in accordance with the International Council for Harmonisation Good Clinical Practice and the guiding principles of the Declaration of Helsinki, with approval by institutional review boards or independent ethics committees/health authorities (approval number of Shionogi ethical committee: 14-08, date: December 14, 2013). Participants provided written informed consent. The study protocol is available in Supplementary Material.

### Participants

Participants with advanced or metastatic urothelial carcinoma who had been considered a complete response (CR), partial response (PR), or stable disease (SD) based on the response evaluation criteria in solid tumors (RECIST) version 1.1 at the end of at least four cycles of first-line platinum-containing chemotherapy were eligible. Participants were required to have an Eastern Cooperative Oncology Group performance status (ECOG PS) of 0 or 1, and adequate hematological, renal, and hepatic function. Participants who had progressive disease (PD) on RECIST version 1.1 or who were judged to have clinically progressive symptoms during first-line platinum-containing chemotherapy were excluded.

### Outcome measures and assessments

The primary efficacy endpoint was the CTL induction rate within 12 weeks after initial dose, defined as the proportion of participants showing CTL induction to at least one of the five antigens. CTL induction was defined as an increase in CTL activity at any point after baseline (the CTL activity measurement taken before dosing on the date of the first dose).

Secondary endpoints included CTL induction rate within 1 year after initial dose, response rate (the proportion of participants who are assessed as CR or PR), disease control rate (DCR; the proportion of participants who were assessed as CR, PR, SD, or non-CR/non-PD), any response rate in image analyses such as tumor cavitation, PFS, OS, and change in quality of life (QoL).

Computed tomography (CT) or magnetic resonance imaging (MRI) scans were performed at baseline and every 12 weeks thereafter in participants in the S-588410 and observation groups. Tumor evaluation was performed by central review based on RECIST version 1.1 and immune-related response criteria (irRC) [28]. PFS was defined as the time interval from the date of enrollment to the date of progression (in order of the following priority: PD by central review based on RECIST version 1.1, withdrawal due to aggravation of the target disease, or progression of the target disease by the investigator in the vital status follow-up form) or death due to any cause, whichever occurred first, or the date of last evaluation of progression. OS was defined as the time interval from the date of enrollment to the date of death due to any cause, or the date of last follow-up.

Safety was evaluated in both groups during the study. Adverse events (AEs) and treatment-emergent AEs (TEAEs), defined as AEs reported after the initial dose of study drug in the S-588410 group, were reported and graded according to the CTCAE version 4.03 and coded using MedDRA version 17.0.

Participant QoL was assessed using the European Organization for Research and Treatment of Cancer quality of life questionnaire C30 (EORTC QLQ-C30) and the EuroQol-5 Dimension 5-level version (EQ-5D-5L) questionnaires. Change in QoL was measured from baseline (the value obtained on week 0 before dosing) in the general health status (GHS)/QoL score on the EORTC QLQ-C30 questionnaire, and the index value and the EuroQol visual analog scale (EQ VAS) on the EQ-5D-5L questionnaire, respectively.

Follow-up assessments were performed in all enrolled participants for 3 years after enrollment of the last participant.

### Immunohistochemistry

Expression levels for DEPDC1, MPHOSPH1, URLC10, CDCA1, KOC1, PD-L1, and HLA-class I were measured by immunohistochemistry in archival tumor tissue or biopsies at screening. Tumor site was confirmed by hematoxylin and eosin staining. The five antigens were stained and evaluated as previously described [27]. PD-L1 was stained using the rabbit anti-human PD-L1/CD274 monoclonal antibody (clone: SP142, Spring Bioscience, Inc., Pleasanton, CA). Antigen expression in the tumor cells and tumor-infiltrating immune cells were scored by two researchers as follows: proportion score (PS) = 0: < 1%, 1: 1–5%, 2: 5–10%, 3: > 10%; intensity score (IS) = 0: negative, 1: weak, 2: moderate, 3: strong; determination = positive: PS and IS score≥1; negative: PS or IS score 0. Where there were insufficient numbers of specimens for analysis, “no results” were recorded.

### Immune response monitoring

Peripheral blood mononuclear cells (PBMCs) were obtained from participants in the S-588410 group before vaccination (baseline) and at 8, 12, 24, 36, 48, and 104 weeks and stored at -80°C until used. Immunological response (CTL induction) for each of the five peptides was assessed by enzyme-linked immunospot (ELISPOT) assay after *in vitro* stimulation of PBMCs with peptide [24, 29]. The results of the CTL activity were provided based on interferon-γ production quantity, with (–) denoting no specific CTL activity, (+) specific CTL activity, (++) strong specific CTL activity, and (+++) extremely strong specific CTL activity.

### Statistics

Assuming a CTL induction rate of 75% as determination of sample size, 35 participants were required to have a power of 90% to reject the null hypothesis that the CTL induction rate was equal to or less than 50% by the binomial test at a one-sided significance level of 5%. In anticipation of dropout of 15%, the total target sample size was 42 participants in the S-588410 group. The maximum sample size of 42 participants was required for the observation group to enable the estimation with a similar accuracy to the S-588410 group for PFS and OS as exploratory analyses.

The CTL induction rate within 12 weeks after initial dose (primary endpoint) was assessed in the modified intention-to-treat (mITT) population, defined as all enrolled participants who had a CTL activity measurement at baseline and at least one CTL activity measurement after the initiation of study drug administration. A one-sided binomial test for the null hypothesis that the proportion of participants with CTL induction within 12 weeks was equal to or less than 0.5 was performed at a significance level of 0.05 for the S-588410 group.

Secondary endpoints were assessed in the intention-to-treat (ITT) population (all enrolled participants). Tumor response was summarized using descriptive statistics, and PFS and OS curves were estimated using Kaplan–Meier method in each group.

Safety was evaluated in all enrolled participants in the S-588410 group who received at least one dose of S-588410 and all participants in the observation group (safety population).

Statistical analyses were carried out using SAS software version 9.2 (SAS institute).

## RESULTS

**Fig. 1:**
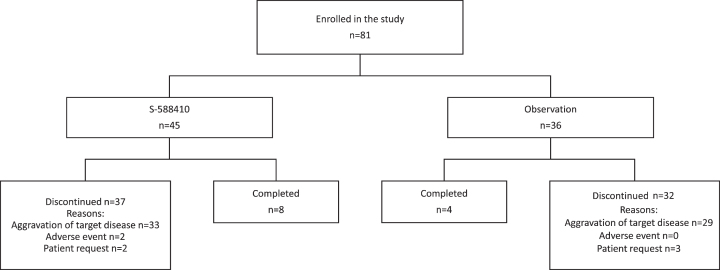
Patient disposition.

A total of 81 participants were enrolled; the first was enrolled on April 17, 2014 and the last completed the treatment/observation period on November 21, 2017. In total, 45 participants with at least one HLA-A^*^24:02 allele were allocated to the S-588410 group and the remaining 36 who were HLA-A^*^24:02 negative were allocated to the observation group (ITT population). Eight participants (17.8%) in the S-588410 group and four (11.1%) in the observation group completed the 2-year study period (Fig. 1). The main reason for study discontinuation was aggravation of target disease, occurring in 89% (33/37) and 91% (29/32) participants who discontinued in the S-588410 and observation groups, respectively.

Participant demographics and characteristics are shown in Table 1. In both the S-588410 and observation groups, the mean age of participants was approximately 67 years, and most were male (76.5%; 62/81) and Asian (91.4%; 74/81). A total of 80% participants had primary lesions in the bladder; 30% had visceral metastases or metastases to lymph nodes according to RECIST version 1.1. All participants received and achieved at least SD at the end of the last cycle of the first-line platinum-based therapy (> 90% received GC). At enrollment, metastases according to the TNM classification were found in 50% or more participants in each group (Table 1 and Supplemental Table S1).

**Table 1:** Patient characteristics in the intention-to-treat population

	S-588410 (*n* = 45)	Observation (*n* = 36)
Sex
Male	36 (80.0)	26 (72.2)
Female	9 (20.0)	10 (27.8)
Age (years), mean±standard deviation	66.7±9.0	67.4±9.3
Race
Asian	44 (97.8)	30 (83.3)
White	1 (2.2)	6 (16.7)
ECOG PS
0	33 (73.3)	27 (75.0)
1	12 (26.7)	9 (25.0)
Primary lesion
Bladder	36 (80.0)	28 (77.8)
Renal pelvis	6 (13.3)	3 (8.3)
Ureter	2 (4.4)	3 (8.3)
Urethra	0	0
Concomitant	1 (2.2)	2 (5.6)
Metastasis as per RECIST version 1.1
Liver	1 (2.2)	1 (2.8)
Lung	5 (11.1)	4 (11.1)
Bone	2 (4.4)	0
Lymph node	5 (11.1)	6 (16.7)
First-line platinum-based therapy
GC	41 (91.1)	34 (94.4)
MVAC	2 (4.4)	1 (2.8)
Number of cycles for the first-line chemotherapy, median (range)	4 (3–12)	5 (4–12)
Overall response at the end of the last cycle of the first-line chemotherapy
CR	12 (26.7)	7 (19.4)
PR	20 (44.4)	15 (41.7)
SD	9 (20.0)	10 (27.8)
Non-CR/non-PD	4 (8.9)	4 (11.1)
TNM_T at enrollment
T0	10 (22.2)	7 (19.4)
T1	1 (2.2)	1 (2.8)
T2	3 (6.7)	4 (11.1)
T3	5 (11.1)	4 (11.1)
T4	1 (2.2)	2 (5.6)
Tx	25 (55.6)	18 (50.0)
TNM_N at enrollment
N0	32 (71.1)	25 (69.4)
N1	5 (11.1)	3 (8.3)
N2	8 (17.8)	6 (16.7)
N3	0	2 (5.6)
TNM_M at enrollment
M0	20 (44.4)	17 (47.2)
M1	25 (55.6)	18 (50.0)
Mx	0	1 (2.8)
All five antigens
At least one positive	43 (95.6)	30 (83.3)
All negative	1 (2.2)	1 (2.8)
All no results	1 (2.2)	5 (13.9)
DEPDC1
Positive	42 (93.3)	28 (77.8)
Negative	2 (4.4)	3 (8.3)
No result	1 (2.2)	5 (13.9)
MPHOSPH1
Positive	42 (93.3)	30 (83.3)
Negative	1 (2.2)	1 (2.8)
No result	2 (4.4)	5 (13.9)
URLC10
Positive	35 (77.8)	23 (63.9)
Negative	8 (17.8)	8 (22.2)
No result	2 (4.4)	5 (13.9)
CDCA1
Positive	35 (77.8)	26 (72.2)
Negative	8 (17.8)	5 (13.9)
No result	2 (4.4)	5 (13.9)
KOC1
Positive	33 (73.3)	24 (66.7)
Negative	10 (22.2)	7 (19.4)
No result	2 (4.4)	5 (13.9)
HLA class I
Positive	43 (95.6)	31 (86.1)
Negative	0	0
No result	2 (4.4)	5 (13.9)
PD-L1 in tumor cells
Positive	9 (20.0)	6 (16.7)
Negative	30 (66.7)	19 (52.8)
No result	6 (13.3)	11 (30.6)
PD-L1 in tumor-infiltrating immune cells
Positive	17 (37.8)	10 (27.8)
Negative	22 (48.9)	15 (41.7)
No result	6 (13.3)	11 (30.6)

Data are *n* (%) unless otherwise stated. Abbreviations: CDCA1 =cell division cycle associated 1; CR = complete response; DEPDC1 = DEP domain containing 1; ECOG PS = Eastern Cooperative Oncology Group performance status; GC = gemcitabine and cisplatin; HLA = human leukocyte antigen; KOC1 = KH domain-containing protein overexpressed in cancer 1; MPHOSPH1 = M-phase phosphoprotein 1; MVAC = methotrexate, vinblastine, adriamycin and cisplatin; PD-L1 = programmed death ligand 1; PR =partial response; SD = stable disease; URLC10 = up-regulated lung cancer 10.

Expression of any one of the five antigens and HLA class I was found in the tumor tissue of most participants in the S-588410 (95.6% [43/45] and 95.6% [43/45], respectively) and observation groups (83.3% [30/36] and 86.1% [31/36], respectively). The proportion of participants with tumor PD-L1 expression in tumor and in tumor-infiltrating cells was comparable between the S-588410 (20.0% [9/45] and 37.8% [17/45], respectively) and observation (16.7% [6/36] and 27.8% [10/36], respectively) groups. Participant demographics and characteristics were similar between groups.

The rate of CTL induction in participants to any of the five antigens at least once during the first 12 weeks of treatment was 93.3% (42/45 in mITT population) and the null hypothesis that the CTL induction rate for 12 weeks would be 50% or less was rejected significantly (*p* < 0.0001, one-sided binomial test).

**Fig. 2:**
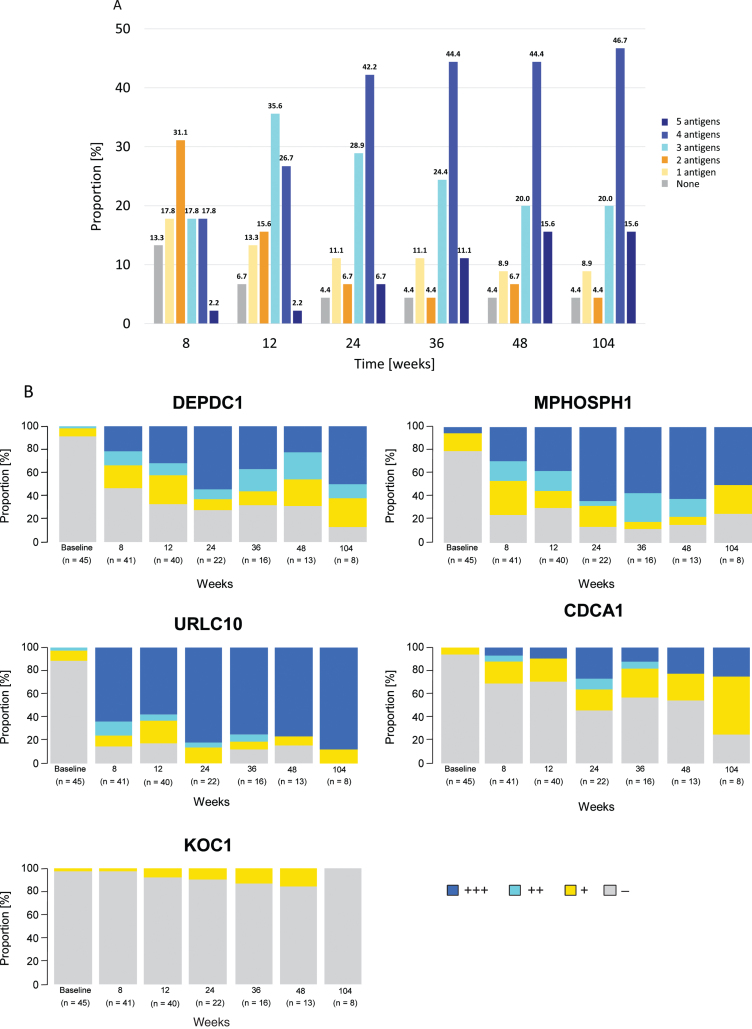
Time-dependent dynamics of cytotoxic T-lymphocyte (CTL) induction. (A) Proportion of patients by number of antigens (0–5) exhibiting CTL induction at least once within each time point relative to the total (*n* = 45). CTL induction of each antigen is defined as increased corresponding CTL grade compared with baseline at any point from the start of study drug administration. (B) Percentage of patients by CTL grades (-, +, ++, +++) for each peptide at the time point. Number of patients at each time point were 45, 41, 40, 22, 16, 13, and eight at baseline, 8, 12, 24, 36, 48, and 104 weeks, respectively. Abbreviations: CDCA1 = cell division cycle associated 1; DEPDC1 = DEP domain containing 1; KOC1 = KH domain-containing protein overexpressed in cancer 1; MPHOSPH1 = M-phase phosphoprotein 1; URLC10 = up-regulated lung cancer 10.

A steady increase in CTL induction in participants to any of the five antigens was observed, with induction rates of 86.7% (39/45) at week 8 and 95.6% (43/45) at weeks 24 and 48. The highest proportion of participants by number of antigens exhibiting CTL induction at least once within each time point was: two antigens at 8 weeks (31.1%, 14/45), three antigens at 12 weeks (35.6%, 16/45), and four antigens after 24 weeks (from 42.2%, 19/45 at 24 weeks to 46.7%, 21/45 at 104 weeks) (Fig. 2A). After 12 weeks, CTL induction rates (90% confidence interval [CI]) were 69.0% (55.4–80.6%) and 80.0% (66.8–89.6%) for DEPDC1 (in 29/42 evaluable participants) and MPHOSPH1 (in 32/40 evaluable participants), respectively; these were 88.6% (75.7–96.0%), 62.9% (47.6–76.4%) and 21.2% (10.4–36.2%) for URLC10 (in 31/35 evaluable participants), CDCA1 (in 22/35 evaluable participants), and KOC1 (in 7/33 evaluable participants), respectively, at plateau. CTL activities by grade for each peptide at the time points measured are shown in Fig. 2B. The probability of a higher grade of CTL activity was increased in a time-dependent manner.

The antitumor response rate in participants was the same whether it was assessed by irRC (Table 2) or RECIST version 1.1 (Supplemental Table S2) and was 8.9% (4/45) in the S-588410 group and 0% (0/36) in the observation group. The DCR was 22.2% (10/45) as assessed by irRC and 24.4% (11/45) as assessed by RECIST version 1.1 in the S-588410 group and was 13.9% (5/36) as assessed by both methods in the observation group.

**Table 2:** Antitumor response assessed by immune-related response criteria

	S-588410 (*n* = 45)	Observation (*n* = 36)
Best overall response
irCR	1 (2.2)	0
irPR	3 (6.7)	0
irSD	6 (13.3)	5 (11.1)
irPD	8 (17.8)	5 (11.1)
No disease	9 (20.0)	13 (28.9)
Not evaluable	18 (40.0)	13 (28.9)
Response rate (irCR + irPR)	4 (8.9)	0
90% CI	(3.1–19.2)	(0.0–8.0)
Disease control rate (irCR + irPR + irSD)	10 (22.2)	5 (13.9)
90% CI	(12.6–34.8)	(5.6–27.0)

Data are *n* (%). Abbreviations: CI = confidence interval; irCR =immune-related complete response; irPD = immune-related progressive disease; irPR = immune-related partial response; irRC =immune-related response criteria; irSD = immune-related stable disease.

**Fig. 3:**
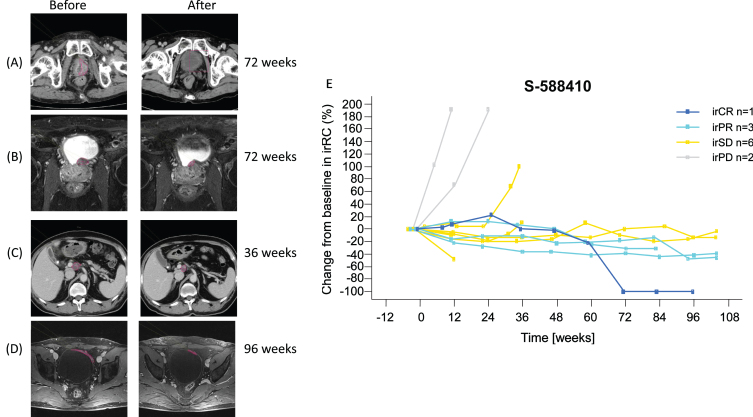
Objective response in four participants. (A) Computed tomography image shows tumor in the bladder at screening, as indicated by the red line. After vaccination for 72 weeks, the tumor regressed and was judged as a complete response (CR). (B) Pelvic MRI shows tumor in bladder posterior at screening, as indicated by the red line. After vaccination for 72 weeks, the tumor shrank and was judged as a partial response (PR). (C) Computed tomography image shows progression of metastasis in abdominal node(s) porta hepatis at screening, as indicated by the red line. After vaccination for 36 weeks, the tumor shrank and was judged as a PR. (D) Pelvic MRI shows tumor in bladder at screening, as indicated by the red line. After vaccination for 96 weeks, the tumor shrank and was judged as PR. (E) Change in target lesion assessed by irRC. Six irPD participants in the S-588410 group are not shown due to no target lesion being identified prior to the first dose. Abbreviations: irCR = immune-related complete response; irPD = immune-related progressive disease; irPR = immune-related partial response; irRC = immune-related response criteria; irSD = immune-related stable disease.

Tumor imaging showed PR in three participants and CR in one participant after at least 36 weeks (Fig. 3A–D). Gradual (PR, *n* = 3) and durable (CR, *n* = 1) tumor shrinkage in the target lesions were shown in the S-588410 group (Fig. 3E), but not in the observation group (Supplemental Fig. S2).

**Fig. 4:**
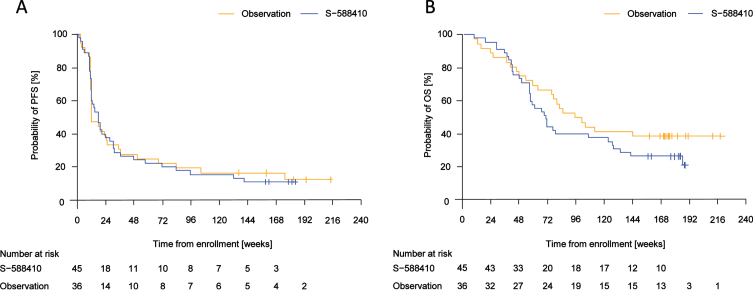
Kaplan–Meier estimates of progression-free survival (PFS) and overall survival (OS). (A) Median (range of event time) PFS was 18.1 (2.1–141.7) weeks in the S-588410 group and 12.5 (3.4–176.1) weeks in the observation group. (B) Median (range of death time) OS was 71.0 (8.9–188.7) weeks in the S-588410 group and 99.0 (8.7–145.7) weeks in the observation group.

Median (range of event time) PFS time was 18.1 (2.1–141.7) weeks in the S-588410 group and 12.5 (3.4–176.1) weeks in the observation group (Fig. 4A). Median (range) OS time was 71.0 (8.9–188.7) weeks in the S-588410 group and 99.0 (8.7–145.7) weeks in the observation group (Fig. 4B).

The proportion of participants who received at least one subsequent therapy other than surgery for the target disease was 73% (33/45) in the S-588410 group and 75% (27/36) in the observation group. Of these participants, 52% (17/33) in the S-588410 group and 48% (13/27) in the observation group received GC therapy, 12% (4/33) in the S-588410 and 11% (3/27) in the observation group received MVAC therapy, and 82% (27/33) in S-588410 and 93% (25/27) in the observation group received other therapy including immune checkpoint inhibitors.

In total, 334 TEAEs/AEs were reported, comprising 225 TEAEs in 97.8% (44/45) of participants in the S-588410 group and 109 AEs in 61.1% (22/36) of participants in the observation group. The incidence of AEs by maximum severity in the S-588410 and observation groups were grade 1–2 in 68.9% (31/45) and 47.2% (17/36) of participants, respectively, and grade 3 in 26.7% (12/45) and 13.9% (5/36) of participants, respectively. A total of 13 serious AEs were reported in 13.3% of participants (6/45) in the S-588410 group. Of these only one serious TEAE in a single participant, which was associated with interstitial lung disease (grade 3), was considered related to S-588410 because the shadow of disease appeared after the initiation of treatment and resolved after S-588410 was discontinued. In the S-588410 group, 4.4% (2/45) withdrew from the study due to TEAEs: one participant with serious pneumonia (grade 3), which was ongoing after discontinuation, and one owing to serious malignant neoplasm progression, from which the participant died. These events were not considered to be related to S-588410.

AEs and TEAEs by MedDRA preferred term are summarized in Table 3. The most frequent TEAEs in the S-588410 group were injection site reactions (47 events), which were reported in 93.3% (42/45) of participants including 4.4% (2/45) with grade 1, 80.0% grade 2 (36/45) and 8.9% (4/45) grade 3 injection-site reactions. In addition, pyrexia 20.0% (9/45), rash 8.9% (4/45), and pruritus 6.7% (3/45) of participants were detected in the S-588410 group, but not in the observation group.

**Table 3:** TEAEs for S-588410 and AEs for the observation group (incidence ≥5% each in the group)

	S-588410 (*n* = 45)	Observation (*n* = 36)
General disorders and administration-site conditions
Injection-site reaction	42 (93.3)	0
Pyrexia	9 (20)	0
Malaise	3 (6.7)	0
Fatigue	1 (2.2)	2 (5.6)
Skin and subcutaneous tissue disorders
Rash	4 (8.9)	0
Dermatitis contact	3 (6.7)	0
Pruritus	3 (6.7)	0
Infections and infestations
Nasopharyngitis	6 (13.3)	2 (5.6)
Urinary tract infection	0	4 (11.1)
Gingivitis	0	2 (5.6)
Helicobacter infection	0	2 (5.6)
Gastrointestinal disorders
Vomiting	3 (6.7)	3 (8.3)
Constipation	3 (6.7)	1 (2.8)
Nausea	3 (6.7)	0
Abdominal pain	0	4 (11.1)
Dental caries	0	2 (5.6)
Dyspepsia	0	2 (5.6)
Psychiatric disorders
Insomnia	3 (6.7)	1 (2.8)
Nervous system disorder
Headache	3 (6.7)	2 (5.6)
Neuropathy peripheral	0	2 (5.6)
Vascular disorders
Hypertension	3 (6.7)	2 (5.6)
Deep vein thrombosis	0	2 (5.6)
Renal and urinary disorders
Dysuria	2 (4.4)	2 (5.6)
Hematuria	2 (4.4)	2 (5.6)
Musculoskeletal and connective tissue disorders
Back pain	1 (2.2)	4 (11.1)
Pain in extremity	1 (2.2)	2 (5.6)
Musculoskeletal pain	0	3 (8.3)

Data are *n* (%). Abbreviations: AE = adverse event; TEAE = treatment-emergent adverse event.

No clinically significant changes in laboratory tests (hematology, blood chemistry, urinalysis), vital signs (blood pressure, pulse rate, body temperature), or electrocardiogram findings were observed in the S-588410 group at any point during the study.

The mean±standard deviation change from baseline in the EORTC QLQ-C30 scores, EQ-5D-5L index value, and EQ VAS scores were –21.7±24.8, –0.1292±0.1814, and –12.7±20.6, respectively, in the S-588410 group at the last observation and were –3.8±14.3, –0.0322±0.1652, and –2.6±15.7, respectively, in the observation group at the last observation. Mean change in these scores during the study initially deteriorated, but mild recovery was observed after 36 weeks in the S-588410 group (Supplemental Fig. S3).

## DISCUSSION

This phase 2 study was the first to investigate the immune response to, and efficacy and safety of, the cancer peptide vaccine S-588410 as maintenance monotherapy after first-line platinum-containing chemotherapy in patients with advanced or metastatic urothelial carcinoma. This immunotherapy was restricted to HLA-A^*^24:02-individuals (more common in Japan) using peptides derived from five cancer-testis antigens (DEPDC1, MPHOSPH1, URLC10, CDCA1, and KOC1) commonly present in bladder cancer. In this study, high rates of CTL induction towards these peptides were achieved over a 2-year period in participants whose diseases remained stable after completion of first-line platinum-containing chemotherapy (predominantly GC).

S-588410 has previously demonstrated successful CTL induction against each of the five peptides, particularly URLC10, following a median of five doses (range: 3–14) in a phase 1 study in esophageal cancer [27]. In the present study, we demonstrated that as the number of doses increased, a higher proportion of patients showed multi-peptide CTL induction. High CTL induction was maintained by biweekly vaccination up to 24 months in the eight participants who had completed S-588410 treatment, suggesting immune tolerance of these peptides could not be induced by repeated vaccination of S-588410.

The results from the present study indicate that CTL induction of each peptide in S-588410 is at a similar level to those achieved in earlier studies. For example, CTL induction against DEPDC1 and MPHOSPH1 has been previously observed in patients with bladder cancer in response to a cancer vaccine comprising two peptides administered weekly for 12 weeks [24, 25]. Specifically, the CTL induction rate against either DEPDC1 or MPHOSPH1 was 88.9% after 12 weeks of treatment [24]. Furthermore, DEPDC1 or MPHOSPH1 peptide-specific CTL responses were observed in 77.5% or 75.8% of patients with non-muscle-invasive bladder cancer after 11 doses of vaccine were administered in combination with intravesical BCG therapy [25]. In the current study, we observed CTL induction rates (90% CI) of 69.0% (55.4–80.6%) and 80.0% (66.8–89.6%) for DEPDC1 and MPHOSPH1, respectively, after 12 weeks. Furthermore, lymphocyte antigen 6 family member K (LY6K)- (also known as URLC10), CDCA1-, and U3 small nucleolar ribonucleoprotein protein IMP3 (IMP3)- (also known as KOC1) specific CTL responses of 85.7%, 64.3%, and 42.9% have been identified previously in patients with advanced head and neck squamous cell carcinoma [30]. We observed comparable CTL induction rates (90% CI) of URLC10, CDCA1, and KOC1 in 88.6% (75.7–96.0%), 62.9% (47.6–76.4%), and 21.1% (10.4–36.2%) of patients at plateau in the present study.

In the present study, gradual (immune-related PR, *n* = 3) and durable (immune-related CR, *n* = 1) tumor shrinkage was shown after at least 36 weeks in the S-588410 group. Participants who experienced an antitumor response appeared to have induced CTL activity for DEPDC1, MPHOSPH1, and URLC10 and prolonged survival. High expression of DEPDC1 and MPHOSPH1 was detected by immunohistochemical analysis in around 90% of participants with bladder cancer, similar to previous studies [24, 25]. On the other hand, slightly fewer participants were positive for the expression of URLC10 (72%), CDCA1 (75%), and KOC1 (70%) than DEPDC1 and MPHOSPH1. High expression of cancer-testis antigens in tumors suggest the possibility that the antigen-specific CTL could infiltrate from the circulatory system into tumor as TIL by S-588410 vaccination. In previous bladder cancer studies, CTL induction was associated with longer recurrence-free survival or OS than in patients without a CTL response [24, 25]. However, no obvious association was found between efficacy and the status of CTL induction in our study. Whilst the CTL induction rate was high in patients receiving S-588410, the antitumor response was low. There may be specific factors related to antitumor response in those patients whose tumor growth was suppressed. However, expression of tumor immunology-related molecules was not assessed after vaccination, therefore, it is unclear why a disconnect between immunological response and clinical objective response was seen in patients treated with S-588410.

There were some limitations to this study. First, the small sample size could affect interpretation of the data, particularly survival evaluation. Consequently, an analysis of significance test for survival differences between the two groups was not planned. However, the Kaplan–Meier curves indicate that OS appeared to be longer for participants in the observational than the treatment group. This is unlikely to be due to differences between the groups in subsequent therapy received as the number and types of therapy were similar between the groups. Second, many participants were Tx or T0 according to the TNM classification at enrollment (Table 1 and Supplemental Table S1); this was also the case among the 12 participants who completed the study (Supplemental Table S3). This may have affected the evaluation of antitumor response. Furthermore, the open-label design of the study could have led to some differences in the timing of the start of follow-up. Another limitation of the study is that participants were not randomized to treatment or observation, but instead were selected by HLA-A genotype.

Antigenic peptides, including those in S-588410, have also been studied in non-randomized phase 2 trials in several cancers using an HLA-key open study design in Japan; that is, vaccination to all enrolled patients without knowing HLA-A genotype status until analysis. The survival efficacy data for these peptide vaccinations in these studies were inconclusive [25, 29–31]. In patients with esophageal squamous cell carcinoma treated with HLA-A24 binding peptides from TTK protein kinase, LY6K, and IMP3, OS was longer, but not significantly, in patients who were HLA-A^*^24:02 positive (A24[+]) compared with those who were HLA-A^*^24:02 negative (A24[–]); median survival time was 4.6 versus 2.6 months (*p* = 0.121) [29]. Similarly, OS was not significantly different in patients with advanced gastric cancer treated with multiple peptides (DEPDC1, URLC10, FoxM1, Kif20A, and VEGFR1) who were A24(+) compared with A24(–) [31]. However, OS was significantly longer in patients with advanced head and neck cancer treated with LY6K, CDCA1, and IMP3 peptides who were A24(+) compared with those who were A24(–); median survival time was 4.9 versus 3.5 months; *p* < 0.05) [30]. In the present study, most of the participants were Japanese as the HLA-A^*^24:02 genotype is more common in Japan than in Europe. Moreover, a higher proportion of white participants with HLA-A^*^24:02 negative genotypes were enrolled in the observation group than the S-588410 group (16% versus 2%). These differences may reflect differences in OS reported between the treatment and observation groups in this study, although there is no evidence to support this. Therefore, placebo-controlled randomized trials are needed to exclude any potential influence of differences in the HLA genotype of participants in the vaccine group and control groups seen in single-arm studies. A phase 3 randomized controlled trial of S-588410 in HLA-A^*^24:02 genotype esophageal cancer patients is currently underway (UMIN000016954).

Recently, maintenance therapy with pembrolizumab significantly improved PFS versus placebo in the phase 2 HCRN GU14-182 study of patients with SD following eight or fewer cycles of platinum-based chemotherapy [32]. Furthermore, avelumab plus best supportive care offered superior OS versus best supportive care alone (median OS 21.4 versus 14.3 months; HR 0.69; 95% CI 0.5–0.86; *p* = 0.001) as maintenance therapy after first-line platinum-based chemotherapy in analysis of the phase 3 JAVELIN Bladder 100 study [33]. In addition, OS was improved after avelumab maintenance therapy in patients whose tumors expressed PD-L1 in that study [33]. PD-L1 expression has also been correlated with urothelial carcinoma severity and outcome of treatment with immune checkpoint inhibitors [34]. S-588410 was previously reported to induce peptide-specific functional CD8(+) cells and PD-L1 expression in esophageal tumors [27]. Furthermore, 5-year survival in esophageal cancer patients treated with a cancer peptide vaccine including URLC10, CDCA1, and KOC1 was significantly higher than in untreated patients (68.0% versus 17.7%; *p* = 0.010) in the sub-population defined as CD8(–)/PD-L1(–) [35]. If the observed increases in intratumoral CD8(+) cells and PD-L1 expression with S-588410 occur in patients with urothelial carcinoma, combination therapy with S-588410 and an anti-PD-L1 antibody may offer an effective treatment option. However, there is currently no evidence to support this hypothesis. Although it is generally believed that it takes time to acquire immune response, the finding that high CTL induction was shown after 8 weeks of administration could provide evidence for the benefit of starting S-588410 treatment immediately after first-line chemotherapy instead of waiting for disease progression. To investigate this approach, a phase 1b trial of vaccine peptides plus nivolumab conducted in bladder and other cancers [36], and a phase 1b/2 study in non-muscle invasive bladder cancer (NCT04106115) are being conducted. Although it may be necessary to scrutinize the administration conditions, including the administration interval and the optimal timing for ending the cancer peptide vaccine and for starting the immune checkpoint inhibitor, cancer peptide vaccines may have the potential to offer additional clinical benefit if combined with immune checkpoint inhibitor in this setting.

In previous clinical studies of cancer peptide vaccines, injection-site reactions were considered to be the most frequent TEAEs [24–27, 29–31]. In the current study, injection-site reactions were reported in 93.3% of participants, mostly grade 2 (80.0%).

In conclusion, S-588410 induced a strong and sustained CTL response in patients with SD after first-line platinum-based therapy for advanced or metastatic urothelial carcinoma. Injection-site reactions were the most common TEAEs related to S-588410, and no new safety issues specific to S-588410 were found. Although S-588410 showed modest clinical response in terms of antitumor evaluation, further studies are needed to confirm antitumor response and survival benefit as maintenance therapy.

## Supplementary Material

Supplementary Material Clinical Study Protocol.

Supplementary Tables

Supplementary Figure 1 Study design.

Supplementary Figure 2A Change in target lesion assessed by irRC.

Supplementary Figure 2B

Supplementary Figure 3A-D Change from baseline (the value obtained at week 0 before dosing) in the in intention-to-treat population in the EORTC QLQ-C30 global health status/QoL score, EQ-5D-5L index value, and EQ VAS.

Supplementary Figure 3E

Supplementary Figure 4

## DATA SHARING STATEMENT

Data sharing from this study may be available on reasonable request by healthcare providers, investigators, or researchers to address specific scientific or clinical research objectives. Shionogi is committed to reviewing requests from researchers for access to de-identified patient-level clinical trial data, and study-level clinical trial data. See more: https://www.shionogi.com/global/en/company/policies/clinical-trial-data-transparency-policy.html
