# Supplementary material for: A Phase 2 Study of S-588410 Maintenance Monotherapy for Platinum-Treated Advanced or Metastatic Urothelial Carcinoma
Source: Bladder Cancer. 2022 Jun 3;8(2):179–92. doi: 10.3233/BLC-211592 (PMC11181746; doi:10.3233/BLC-211592)
Supplement: Supplementary Material — Clinical Study Protocol. [file blc-8-blc211592-s001.pdf]

Clinical Study Protocol: 1408P1921

**Study Title:**	A multicenter, open-label, phase 2 study of S-588410 as maintenance monotherapy after first-line platinum-containing chemotherapy in patients with advanced and/or metastatic bladder cancer
**Study Number:**	1408P1921
**Eudra CT number:**	2013-005274-22
**Study Phase:**	2
**Product Name:**	S-588410
**Sponsor:**	Shionogi & Co., Ltd.
**Sponsor Contact:**	Clinical Research Department, Shionogi & Co., Ltd.<br>12th floor, Hankyu Terminal Bldg., 1-4, Shibata 1 - chome, Kita-ku, Osaka 530-0012, Japan<br>TEL: [redacted]
	Shionogi Ltd.<br>5th floor, 33 Kingsway, Holborn, London<br>WC2B 6UF, United Kingdom<br>TEL: [redacted]

Issue Date:

**Original:**	25 December 2013
**Amendment 1:**	24 January 2014
**Amendment 2:**	18 March 2014
**Amendment 3:**	19 January 2015
**Amendment 4:**	11 March 2015
**Amendment 5:**	3 October 2016
**Amendment 6:**	21 February 2017
**Amendment 7:**	1 April 2017

Confidentiality Statement

This document and the information contained herein or attached hereto ("Confidential Material") are confidential and proprietary to Shionogi & Co., Ltd. This Confidential Material should only be viewed by those individuals or companies that have been given prior written authorization to do so by Shionogi & Co., Ltd. ("Authorized Users"). This Confidential Material should not be made available in any form to any person or company, other than the Authorized Users and their respective employees or associates on a need-to-know basis, without the written consent from Shionogi & Co., Ltd.

SYNOPSIS

Study Title:

A multicenter, open-label, phase 2 study of S-588410 as maintenance monotherapy after first-line platinum-containing chemotherapy in patients with advanced and/or metastatic bladder cancer

Study Number: 1408P1921

Study Phase: 2

Objectives:

The primary objective is:

- To evaluate the specific cytotoxic T lymphocyte (CTL) response in human leukocyte antigen (HLA)-A*24:02-positive patients receiving S-588410 for 12 weeks.

The secondary objectives are:

- To evaluate the specific CTL induction over time in HLA-A*24:02-positive patients receiving S-588410 for 1 year.
- To estimate antitumor effect in HLA-A*24:02-positive patients receiving S-588410.
- To estimate progression-free survival (PFS) in HLA-A*24:02-positive patients receiving S-588410.
- To estimate overall survival (OS) in HLA-A*24:02-positive patients receiving S-588410.
- To evaluate the safety and tolerability in patients receiving S-588410.
- To assess the general health status in terms of European Organization for Research and Treatment of Cancer (EORTC) QLQ-C30 and Euro-QOL 5 dimension 5 level version (EQ-5D-5L) questionnaires in HLA-A*24:02-positive patients receiving S-588410.
- To collect data of tumor evaluation, PFS, OS, and EORTC QLQ-C30 and EQ-5D-5L questionnaires in HLA-A*24:02-negative patients.

Study Design:

The study is an open-label, multicenter study and consists of 2 study periods:

- The Screening Period is 28 days prior to enrollment. Potential patients who provide written informed consent will be assessed for their eligibility in the screening period.
- The Treatment Period/Observation Period is 24 months (104 weeks) after enrollment. Eligible patients will be enrolled in the study, and assigned to S-588410 Group or Observation Group depending on the HLA-A genotype. Enrollment form will be reported via FAX.
  - HLA-A*24:02-positive patients will be enrolled in the S-588410 Group. Each patient will receive 1 mL of S-588410 emulsion, which contains 1 mg each of S-288301 (DEPDC1-derived peptide), S-288302 (MPHOSPH1-derived peptide), S-488401 (URLC10-derived peptide), S-488402 (CDCA1-derived peptide), and S-488403 (KOC1-derived peptide). S-588410 will be injected subcutaneously in the patient's inguinal, axillary, or cervical region once weekly for 12 weeks and once every 2 weeks thereafter for up to 24 months. They will undergo efficacy assessments (specific CTL measurement, anti-tumor evaluation, PFS, OS, and EORTC QLQ-C30 and EQ-5D-5L questionnaire assessment), safety assessments (injection site assessment, physical examination, vital sign assessment,

electrocardiogram [ECG], Eastern Cooperative Oncology Group Performance Status [ECOG PS], laboratory tests, and adverse event [AE] monitoring), and other assessments (antigen expression testing and T-cell receptor repertoire analysis).

- HLA-A*24:02-negative patients will be enrolled in the Observation Group and will not receive the study drug. They will undergo the efficacy and safety assessments similarly to the S-588410 Group except for specific CTL measurement, injection site assessment, and T-cell receptor repertoire analysis.

Follow-up assessments will be performed in all enrolled patients up to 3 years after enrollment of the last patient. The following data will be reported to the sponsor: survival or death, date and site of progression, and date, description of, and response to subsequent therapy for the target disease (ie, bladder cancer).

Study Population:

Male or female patients 20 years of age or older, with advanced and/or metastatic bladder cancer.

Inclusion Criteria:

Patients who fulfill all of the following criteria assessed by the end of screening period will be included in the study.

1. Patients with advanced and/or metastatic bladder cancer (including urothelial cancer of renal pelvis, ureters, and urethra) who have complete response (CR), partial response (PR), or stable disease (SD) based on the Response Evaluation Criteria in Solid Tumors (RECIST) version 1.1 at the end of at least 4 cycles of first-line platinum-containing systemic chemotherapy.
   (Note: Patients who have subsequently received another platinum-containing chemotherapy due to intolerance* of first-line platinum-containing chemotherapy are eligible if the total number of cycles of platinum-containing chemotherapy reached 4 cycles or more. The patients had no PD at the time of change of platinum-containing chemotherapy. *: any non-hematologic toxicity with grade 3 or higher, or hematologic toxicity with grade 4, according to Common Terminology Criteria for Adverse Events [CTCAE] version 4.03.)
2. Patients who are male or female aged ≥20 years at the time of informed consent.
3. Patients with the ECOG PS 0 or 1 at enrollment.
4. Patients who have a lymphocyte count accounting for 15% or higher of the total white blood cell count within 28 days before enrollment.
5. Patients who are willing and able to provide formalin-fixed and paraffin-embedded bladder cancer tissues for antigen expression measurement.
6. Patients who provide a personally signed and dated informed consent document for participation in the study.

Exclusion Criteria:

Patients who meet any of the following criteria by the end of screening period will be excluded from the study:

1. Patients who have progressive disease (PD) on RECIST version 1.1 at the end of at least 4 cycles of first-line platinum-containing chemotherapy.
2. Patients who are judged to have clinically progressive symptoms at the end of at least 4 cycles of first-line platinum-containing chemotherapy by the investigator or subinvestigator.

3. (deleted)
4. Patients with a history of malignant cancer (except for carcinoma in situ or intra-mucosal cancer that resolved with endoscopic therapy) within 5 years before enrollment.
5. Patients who received any prior therapies for target disease within 3 weeks before the first administration of S-588410.
6. Patients who cannot receive the first administration of S-588410 within 12 weeks after the last administration of chemotherapy.
7. Patients who are expected to require any of the following therapies between enrollment and completion or discontinuation of the study treatment.
   – Anti-malignant tumor drug
   – Systemic corticosteroid (except for corticosteroid defined as the equivalent of prednisone ≤ 10 mg/day orally)
   – Systemic immunosuppressant drug
   – Immunotherapy
   – Radiotherapy (except for restricted radiotherapy for pain relief of bone metastasis) for the target disease
   – Surgical therapy for the target disease
   – Hyperthermia for the target disease
   – Herbal medicine with anti-tumor or immunosuppressant effect
   – Other investigational new products
8. Patients who have severe (CTCAE version 4.03 grade 3 or higher) concurrent hepatic impairment, renal impairment, heart disease, hematological disease, respiratory disease, or metabolic disease, with the exception of any symptoms and/or signs associated with target disease.
9. Patients who have the following laboratory data with grade 3 or higher according to CTCAE version 4.03 criteria within 28 days before enrollment.
   – White blood cell count <2000/mm^3 or >100 000/mm^3
   – Platelet count <50 000/mm^3
   – Hemoglobin <8.0 g/dL
   – Aspartate aminotransferase (AST) or alanine aminotransferase (ALT) >5.0 × the upper limit of normal (ULN)
   – Total bilirubin >3.0 × ULN
   – Serum creatinine >3.0 × ULN
10. Patients who has an increase in an eosinophil counts (more than 5000/mm^3), within 28 days before enrollment, or patients with a history or current complication of eosinophilic pneumonia or interstitial pneumonia. If patients have symptoms and signs suggestive of pneumonia before enrolment, the investigator or subinvestigator must confirm the presence or absence of concurrent eosinophilic pneumonia or interstitial pneumonia on the basis of additional examinations such as chest x-ray.
11. Patients who have known human immunodeficiency virus infection.
12. Patients with uncontrolled systemic or active infection.
13. Patients who had any diseases with the risk of sudden death within 12 months before enrollment.
    [Examples]
    – Myocardial infarction

   - Unstable angina
   - Coronary or peripheral artery bypass graft surgery
   - Thrombotic or embolic events such as pulmonary embolism, deep vein thrombosis, or transient ischemic attack
14. Patients who have known brain metastases.
15. Patients with a history or evidence of autoimmune diseases and/or immunodeficiency disorders.

    [Examples]
    - Systemic lupus erythematosus
    - Multiple sclerosis
    - Systemic sclerosis
    - Rheumatoid arthritis
16. Patients with a history of severe (CTCAE version 4.03 grade 3 or higher) allergic reaction to a drug, vaccination, or biological preparation.
17. Patients who are hospitalized for treatment at enrollment.
18. Female patients who are lactating or pregnant. Female patients who are of childbearing potential, not included in any of the following, are positive for the pregnancy test at enrollment.
    - Postmenopausal woman (at least 2 years since their last regular menstrual period without any other medical reason)
    - Women who are surgically sterile by hysterectomy and/or bilateral oophorectomy, or by tubal ligation
19. Patients who cannot or do not intend to practice effective contraception (barrier contraceptives [male condom, female condom, or diaphragm with a spermicidal gel]; hormonal contraceptives [implants, injectables, combination oral contraceptives, or transdermal patches]; or intrauterine devices) as per physicians' recommendations from enrollment to 90 days after completion or discontinuation of study treatment.
20. Patients who received DEPDC1, MPHOSPH1, URLC10, CDCA1, or KOC1 peptide vaccines before.
21. Patients who received any other investigational products within 28 days or 5 half-lives of the investigational products before enrollment whichever is longer.
22. Patients who are considered ineligible for this study by the investigator or subinvestigator due to any reasons, including inability to understand and follow the requirements of the study.

Sample size:

The sample size of the S-588410 Group is 42 patients. The sample size of the Observation Group may be up to 42 patients.

Test Drug, Dose, and Mode of Administration:

The study drug (S-588410) consists of the following 5 peptides: S-288301 (a DEPDC1-derived peptide), S-288302 (a MPHOSPH1-derived peptide), S-488401 (a URLC10-derived peptide), S-488402 (a CDCA1-derived peptide), and S-488403 (a KOC1-derived peptide).

Patients will subcutaneously receive 1 mL of S-588410 emulsion, which includes 1 mg each of 5 peptides, once weekly for 12 weeks and once every 2 weeks thereafter. The study drug will be injected subcutaneously in the patient's inguinal, axillary, or cervical

region. In case that subcutaneous injection in the above region is difficult due to reactions at the injection site, the study drug may be injected in the proximity of the above region to the extent possible.

Control Drug, Dose, and Mode of Administration:

Not applicable

Duration of Treatment:

24 months

Prohibited Concomitant Therapy for Treatment/Observation Period:

- Anti-malignant tumor drug
- Systemic corticosteroid (except for corticosteroid defined as the equivalent of prednisone ≤ 10 mg/day orally)
- Systemic immunosuppressant drug
- Immunotherapy
- Radiotherapy (except palliative radiotherapy for bone metastasis) for the target disease
- Surgical therapy for the target disease
- Thermotherapy for the target disease
- Herbal medicine with anti-tumor or immunosuppressant effect
- Any other investigational new products

Efficacy Assessments:

Specific CTL measurement, anti-tumor evaluation, PFS, OS, and EORTC QLQ-C30 and EQ-5D-5L questionnaire assessment

Safety Assessments:

Assessment of injection site, physical examination, vital signs, ECG, ECOG PS, laboratory tests, and assessment of AEs (CTCAE version 4.03)

Statistical Methods:

Primary endpoint

[S-588410 Group]

- CTL induction rate within 12 weeks after initial dose, defined as the proportion of patients who show in vitro CTL induction to at least any one of the 5 antigens.

Secondary endpoints

[S-588410 Group and Observation Group]

- CTL induction rate within 1 year after initial dose, defined as the proportion of patients who show in vitro CTL induction to at least any one of the 5 antigens.
- Response rate (RR), defined as the proportion of patients who are assessed as CR or PR by using RECIST version 1.1 and immune-related response criteria (irRC), respectively.
- Disease control rate (DCR), defined as the proportion of patients who are assessed as CR, PR, or SD by using RECIST version 1.1 and irRC, respectively.
- Any response rate in image analysis such as tumor cavitation, defined as the proportion of patients who show any tumor change in image analysis (eg, tumor cavitation).
- PFS, defined as the time interval from the date of enrollment to the date of progression (progressive disease based on RECIST version 1.1, clinically progressive symptoms, or withdrawal due to aggravation of the target disease) or death due to any

cause, whichever occurs first.

- OS, defined as the time interval from the date of enrollment to the date of death due to any cause.
- Change in QOL, defined as change from baseline in the global health status, the function scales, and the symptom scales on the EORTC QLQ C30 questionnaire, and the index value and the EQ visual analog scale (VAS) on the EQ-5D questionnaire, respectively. Baseline is defined as the value obtained at Visit 1 (pre-dose).

Efficacy analysis:

Primary endpoint

Primary endpoint will be assessed according to the modified intention-to-treat (mITT) population. As the primary analysis, one-sided binomial test where the null hypothesis is that the CTL induction rate within 12 weeks is equal to 0.5 or less will be performed at a significance level of 0.05. The 90% confidence interval (CI) will then be calculated by using the Clopper-Pearson method.
As a sensitivity analysis, the primary efficacy endpoint will be analyzed using the Per-protocol set (PPS).

Secondary endpoints

For the intention-to-treat (ITT) population, CTL induction rate and the associated 90% CI will be estimated by visit. The 90% CI will then be calculated by using the Clopper-Pearson method. The number of antigens with CTL induction will be tabulated by visit.

For the ITT population, the RR and the DCR using RECIST version 1.1 and irRC will be determined, and the associated 90% CIs will be calculated for the S-588410 Group and the Observation Group, respectively.

For the ITT population, any response rate in image analysis such as tumor cavitation will be determined, and the associated 90% CI will be calculated for the S-588410 Group and the Observation Group, respectively.

For the ITT population, the PFS will be estimated with the Kaplan-Meier method and the median PFS and the associated 90% CI will be presented for the S-588410 Group and the Observation Group, respectively.

For the ITT population, the OS will be estimated with the Kaplan-Meier method and the median OS and the associated 90% CI will be presented for the S-588410 Group and the Observation Group, respectively.

For the ITT population, the change from baseline in the global health status, the function scales, and the symptom scales on the EORTC QLQ C30 questionnaire, and the index value and the EQ VAS on the EQ-5D-5L questionnaire will be summarized by visit for the S-588410 Group and the Observation Group, respectively.

Safety analysis:

AEs will be classified by system organ class and preferred term using Medical Dictionary for Regulatory Activities (MedDRA) Version 16.0 or higher. Of reported AEs on the case report form (CRF), Treatment-emergent adverse events (TEAEs) will be used in the S-588410 Group and AEs will be used in the Observation Group for safety analyses. TEAE is defined as AE occurring after the initial dose of the study drug.

For the S-588410 Group, the number and the percentage of patients who experienced TEAEs will be summarized. For the overall summary of TEAE, the number and the

percentage of patients who have experienced death, serious TEAEs, Grade 3 or higher TEAEs, and TEAEs that lead to withdrawal of study drug will also be summarized. The number of those TEAEs, which are counted by cases reported, will also be presented. Adverse drug reactions (ADRs) will be summarized by the same TEAE category as overall summary.

For the summary of TEAE coded by system organ class and preferred term, the number of patients who have experienced TEAEs will be presented for the S-588410 Group with the percentage of patients. The summary by severity and the summary by outcome will be presented by system organ class and preferred term.

The number and the percentage of patients who experienced local reaction-related AEs at the injection sites will be summarized. In a similar fashion, eosinophilic pneumonia-related AEs, flulike symptoms-related AEs, and anaphylactic reaction-related AEs will be tabulated, respectively.

Study Duration:

Study duration for individual patients:108 weeks (Screening Period, 4 weeks; Treatment Period, 104 weeks)

Planned Overall Study Period: from February 2014 to October 2018

Date of Original: 25 December 2013

TABLE OF CONTENTS

SYNOPSIS....2
TABLE OF CONTENTS....9
LIST OF IN-TEXT TABLES....12
LIST OF ABBREVIATIONS AND DEFINITIONS OF TERMS....13
1. INTRODUCTION....15
  1.1 Bladder Cancer....15
  1.2 Cancer Peptide Vaccine....16
  1.3 Clinical Development of S-588410....16
2. STUDY OBJECTIVES....18
  2.1 Primary Objective....18
  2.2 Secondary Objectives....18
3. INVESTIGATIONAL PLAN....19
  3.1 Overall Study Design and Plan....19
  3.2 Rationale for Study Design....20
    3.2.1 Rationale for the patient population....20
    3.2.2 Rationale for dosage of S-588410....20
  3.3 Study Duration....21
    3.3.1 Study Duration in individual patients....21
    3.3.2 Planned Study Duration for the Study....21
4. STUDY POPULATION SELECTION....22
  4.1 Study Population....22
  4.2 Inclusion Criteria....22
  4.3 Exclusion Criteria....22
5. STUDY TREATMENT(S)....25
  5.1 Description of Treatment(s)....25
    5.1.1 Test Drug....25
    5.1.2 Adjuvant....25
  5.2 Treatments to be Administered....25
    5.2.1 Method for Preparation....25
    5.2.2 Treatments to be Administered....25
  5.3 Selection and Timing of Dose for Each Patient....25
  5.4 Method of Assigning Patients to Treatment Groups....25
  5.5 Blinding....26
  5.6 Management of Clinical Supplies....26
    5.6.1 Study Drug Packaging and Storage....26
    5.6.2 Study Drug Accountability....26
  5.7 Other Supplies....26

6. RESTRICTIONS 27
6.1 Prior Therapy 27
6.1.1 Prior Therapy for the Target Disease 27
6.1.2 Prior Therapy for Other Than the Target Disease 27
6.2 Concomitant Therapy during the Study 27
6.2.1 Prohibited Therapy 28
6.3 Other Provisions 28
7. STUDY PROCEDURES AND METHODS OF ASSESSMENTS 29
7.1 Informed Consent 29
7.2 Baseline Information 29
7.2.1 Baseline Patient Characteristic and Medical History 29
7.2.2 Human Leukocyte Antigen-A Gene Test 30
7.3 Efficacy Assessments 30
7.3.1 Specific Cytotoxic T Lymphocyte Measurement 30
7.3.2 Tumor Evaluation 31
7.3.3 Evaluation of Progression-Free Survival and Overall Survival 31
7.3.4 QOL Assessment 32
7.4 Safety Assessments 32
7.4.1 Assessment of Injection Site 32
7.4.2 Physical Examination 32
7.4.3 Performance Status 33
7.4.4 Vital Signs 33
7.4.5 Electrocardiogram 33
7.4.6 Clinical Laboratory Tests 34
7.5 Other Assessments 35
7.5.1 Antigen Expression Testing 35
7.5.2 T-cell receptor Repertoire Analysis 35
7.6 Adverse Events Assessments 36
7.6.1 Performing Adverse Events Assessments 36
7.6.2 Timing 36
7.6.3 Severity 37
7.6.4 Relationship 37
7.6.5 Expectedness 37
7.6.6 Significant Adverse Events 37
7.6.7 Clinical Laboratory Adverse Events 38
7.6.8 Serious Adverse Events 39
7.6.9 Special Situations-Abuse, Misuse, Overdose, and Medication Error 41
7.6.10 Pregnancy 41

7.6.11 Treatment-Emergent Adverse Events 41
7.7 Withdrawal of Patients from the Study or Study Drug 42
7.8 Appropriateness of Measurements 42
7.9 Acceptable Time Window 43
8. PLANNED STATISTICAL METHODS 45
8.1 General Considerations 45
8.2 Determination of Sample Size 45
8.3 Analysis Populations 45
8.4 Handling of Missing Data 46
8.5 Patient Dispositions 46
8.6 Demographics and Baseline Characteristics 46
8.7 Prior Therapies 46
8.8 Concomitant Therapies 46
8.9 Efficacy Analyses 47
8.9.1 Primary Efficacy Endpoint 47
8.9.2 Secondary Efficacy Endpoints 47
8.9.3 Analyses of Efficacy Endpoints 47
8.10 Safety Analyses 48
8.10.1 Adverse Events 48
8.10.2 Injection Site Reactions 49
8.10.3 Performance Status 49
8.10.4 Vital Signs 49
8.10.5 Electrocardiogram 49
8.10.6 Clinical Laboratory Analysis 50
8.11 Interim Analysis 50
9. ADMINISTRATIVE CONSIDERATIONS 51
9.1 Investigators and Study Administrative Structure 51
9.2 Institutional Review Board or Institutional Ethics Committee Approval 53
9.3 Ethical Conduct of the Study 53
9.4 Patient Information and Consent 53
9.5 Patient Confidentiality 53
9.6 Study Monitoring 54
9.7 Case Report Forms and Source Documents 54
9.7.1 Case Report Forms 54
9.7.2 Source Documents 55
9.7.3 External Data 55
9.8 Committees 55
9.8.1 Case Review Committee 55

9.8.2 Independent Data Monitoring Committee 55
9.9 Termination or Suspension of the Study 56
9.9.1 Termination or Suspension of the Entire Study 56
9.9.2 Termination or Suspension of the Study by Medical Institution 56
9.10 Protocol Deviations and Modifications 56
9.11 Data Management 57
9.12 Retention of Data 57
9.13 Quality Control and Assurance 57
9.14 Publication and Disclosure Policy 58
10. REFERENCE LIST 59
Appendix 1 Time and Events Schedule in the S-588410 Group 61
Appendix 2 Time and Events Schedule in the Observation Group 63
Appendix 3 Management and Discontinuation Criteria for Abnormal Liver Function Tests 65
Appendix 4 EORTC QLQ-C30 Questionnaires 68
Appendix 5 EQ-5D-5L Questionnaires 70
Appendix 6 Vital Status Follow-up Form 72
Appendix 7 Country-Specific Information 77
Japan 77
Appendix 8 Sponsor Signature 78

LIST OF IN-TEXT TABLES

Table 7-1 ECOG PS 33
Table 7-2 Routine Laboratory Tests 34
Table 7-3 Acceptable Time Window 43

LIST OF IN-TEXT FIGURES

Figure 3-1 Study Schematic 20

LIST OF ABBREVIATIONS AND DEFINITIONS OF TERMS

ADR	adverse drug reaction
AE	adverse event
ALP	alkaline phosphatase
ALT	alanine aminotransferase
AST	aspartate aminotransferase
BUN	blood urea nitrogen
CDCA1	cell division cycle associated 1
cDNA	complementary DNA
CI	confidence interval
CK	creatine kinase
CT	computed tomography
CTCAE	Common Terminology Criteria for Adverse Events
CTL	cytotoxic T lymphocyte
CR	complete response
CRF	case report form
CRP	C reactive protein
DEPDC1	DEP domain containing 1
DCR	disease control rate
ECG	electrocardiogram
ECOG	The Eastern Cooperative Oncology Group
ELISPOT	Enzyme-Linked ImmunoSpot
EORTC	European Organization for Research and Treatment of Cancer
EQ-5D-5L	Euro-QOL 5 dimension 5 level version
FDA	Food and Drug Administration
GC	gemcitabine and cisplatin
GCP	Good Clinical Practice
GGT	gamma glutamyl transferase
HLA	human leukocyte antigen
IB	investigator's brochure
ICH	International Conference on Harmonisation
IDMC	independent data monitoring committee
IEC	institutional ethics committee
IRB	institutional review board
irRC	immune-related response criteria

ITT	intention-to-treat
KOC1	KH domain-containing protein overexpressed in cancer 1; alias insulin-like growth factor II mRNA binding protein 3 (IMP-3)
LDH	lactate dehydrogenase
MedDRA	Medical Dictionary for Regulatory Activities
mITT	modified intention-to-treat
MPHOSPH1	M-phase phosphoprotein 1
MRI	magnetic resonance imaging
mRNA	messenger RNA
MVAC	methotrexate, vinblastine, doxorubicin, and cisplatin
OS	overall survival
PBMC	peripheral blood mononuclear cells
PD	progressive disease
PFS	progression-free survival
PPS	per-protocol set
PR	partial response
PS	performance status
RECIST	Response Evaluation Criteria in Solid Tumors
RR	response rate
SAE	serious adverse event
SD	stable disease
TCCU	transitional cell carcinoma of urothelial tract
TCR	T-cell receptor
TEAE	treatment-emergent adverse event
URLC10	up-regulated lung cancer 10; alias lymphocyte antigen 6 complex locus K (LY6K)
ULN	upper limit of normal
VAS	visual analog scale
WHO	World Health Organization

1. INTRODUCTION

1.1 Bladder Cancer

Bladder cancer is the second most common genitourinary malignancy with an incidence of approximately 390,000 new cases and causing 150,000 deaths worldwide in 2008 [1]. Approximately 68,000 patients in the US, 140,000 patients in Europe, and 17,000 patients in Japan were diagnosed with bladder cancer (GLOBOCAN 2008). Approximately 14,000 patients in the US, 55,000 patients in Europe, and 6,000 patients in Japan die from this cancer (GLOBOCAN 2008). The number of patients has increased in recent years (Epidatabase®).

The vast majority (over 90%) of bladder cancers is epithelium carcinomas and the remaining are squamous cell carcinomas and adenocarcinomas. The bladders, the ureters, the urethra, the renal pelvis, and the prostate gland are covered with urothelium. Urothelial carcinoma (also referred to as transitional cell carcinoma) can develop at any place in the organs described above and sometime develop concomitantly. The same chemotherapy is performed for these cancers [2].

Approximately 70% to 80% of the patients are diagnosed as superficial bladder cancer (ie, stage I) [3]. Stage I bladder cancer can often be cured but the risk of recurrence following initial resection is as high as 80% in stage I bladder cancer patients [3].

The standard treatment of muscle invasive bladder cancer (stage III) is preoperative combination chemotherapy and radical cystectomy (including lymph node dissection). However, 5-year progression free survival rate is approximately 75% in patients treated with radical cystectomy and the risk of recurrence is approximately 50% in patients with muscle invasive bladder cancer [3]. The standard treatment of metastatic bladder cancer (stage IV) is systemic chemotherapy with platinum-containing chemotherapy [3, 4]. Widely-used and established regimen as the first-line chemotherapy is a combination of gemcitabine and cisplatin (GC therapy) or a combination of methotrexate, vinblastine, doxorubicin, and cisplatin (MVAC therapy) [3, 4, 5]. Although the initial response rate of GC and MVAC therapies is reported as high (40% to 70%), these regimens are generally not curative (5-year overall survival rate, 4% to 20%; median progression free survival, approximately 8 months; overall survival, approximately 15 months) [6]. No standard second-line therapies are established in metastatic bladder cancer patients in clinical guidelines for bladder cancer in the US, and Japan [3, 5]. In Europe, vinflunine (Jaylor®) is indicated as monotherapy for the treatment of adult patients with advanced or metastatic transitional cell carcinoma of urothelial tract (TCCU) after the failure of a prior platinum-containing regimen. However the efficacy of vinflunine is reported to be modest (overall survival, 6.9 vs. 4.3 months in vinflunine plus best supportive care [BSC] compared with BSC in eligible patients in a phase 3 study) [6, 7] and vinflunine is weakly endorsed in EU clinical guidelines [4, 6]. Therefore, a need remains for effective maintenance therapy, which may postpone the time of progression after completion of first-line chemotherapy.

1.2 Cancer Peptide Vaccine

The cancer peptide vaccine elicits its effect through the cytotoxic T lymphocyte (CTL) induction. The administered epitope peptides are taken up into dendritic cells, which are an antigen presentation cell, and then CTL induction specific to tumor antigen is triggered through the recognition by CTL of the epitope peptide/major histocompatibility complex (MHC) class I on the surface of the cells. In humans, MHC class I is called human leukocytes antigen (HLA) class I. The induced CTL shows cytotoxic activity against target tumor cells of which the epitope peptide/MHC class I is expressed on the surface.

1.3 Clinical Development of S-588410

To date, Shionogi & Co., Ltd. has developed 2 cancer peptide vaccines including the HLA-A*24:02 restricted epitope peptides. These are S-288310 (comprising 2 peptides of S-288301 and S-288302) and S-488410 (comprising 3 peptides of S-488401, S-488402, and S-488403). These peptides are shown as follows:

- S-288301 (DEP domain containing 1 [DEPDC1]-derived peptide)
- S-288302 (M-phase phosphoprotein 1 [MPHOSPH1]-derived peptide)
- S-488401 (up-regulated lung cancer 10, [URLC10]-derived peptide)
- S-488402 (cell division cycle associated 1 [CDCA1]-derived peptide)
- S-488403 (KH domain-containing protein overexpressed in cancer 1 [KOC1]-derived peptide)

Clinical studies of S-288310 have been conducted in patients with bladder cancer since DEPDC1 and MPHOSPH1 were originally found to be expressed in bladder cancer. DEPDC1 and MPHOSPH1 were also found in the upper urothelial carcinoma, and an investigator-initiated translational research study is being conducted in patients with advanced upper urinary tract carcinoma [8]. Clinical studies of S-488410 have been conducted in patients with esophageal cancer or lung cancer since URLC10, CDCA1, and KOC1 were originally found to be expressed in esophageal cancer and lung cancer cells.

Although S-588410 has not been studied in human, the safety and the immunogenicity of these 5 peptides has been already evaluated in these clinical studies of S-288310 or S-488410 (refer to the current investigator's brochure [IB] for detail).

Tumor heterogeneity, tumor immune escape, and difference in immune response (CTL response) to each peptide among patients have been recently recognized through previous development of cancer peptide vaccine and a multipeptide vaccine increases the likelihood of inducing an immune response [9, 10]. Tumor heterogeneity means the difference among patients or tumor cells in antigen expression pattern. Therefore, cancer peptide vaccines including many types of antigen-derived peptide are expected to be highly effective in a wider patient population. All of the 5 antigens have recently been demonstrated to be expressed at high levels on cancer tissues of urinary bladder cancer, esophageal cancer, gastroesophageal cancer, and head and neck cancer tissues (refer to Section 4.1.2 in the current IB for S-588410). Based on these recent findings, the future clinical development will be planned as the HLA-A*24:02 restricted 5-peptide vaccine, S-588410, which includes the five individual peptide of S-288301, S-288302, S-488401, S-488402, and S-488403.

After completion of first-line chemotherapy, maintenance therapy for cancer may prolong the time of progression and result in improvement of overall survival. Maintenance therapy for cancer has already been conducted in clinical studies of some drugs [11, 12, 13], however, none have been approved yet. As a cancer peptide vaccine is expected to have fewer side effects, S-588410 is expected to meet the unmet medical needs of maintenance therapy for patients with advanced and/or metastatic bladder cancer, whose disease responds or remains stable after completion of first-line chemotherapy. Therefore, this open-label, multicenter study with S-588410 as maintenance therapy will be conducted in patients with advanced and/or metastatic bladder cancer whose disease is in complete response (CR), partial response (PR), or stable disease (SD) after completion of first-line chemotherapy as an early phase study (prior to conduct of a large-scale study).

2. STUDY OBJECTIVES

2.1 Primary Objective

The primary objective of this study is:

- To evaluate the CTL induction in HLA-A*24:02-positive patients receiving S-588410 for 12 weeks.

2.2 Secondary Objectives

The secondary objectives of this study are:

- To evaluate the specific CTL induction over time in HLA-A*24:02-positive patients receiving S-588410 for 1 year.
- To estimate antitumor effect in HLA-A*24:02-positive patients receiving S-588410.
- To estimate progression-free survival (PFS) in HLA-A*24:02-positive patients receiving S-588410.
- To estimate overall survival (OS) in HLA-A*24:02-positive patients receiving S-588410.
- To evaluate the safety and tolerability in patients receiving S-588410.
- To assess the general health status in terms of European Organization for Research and Treatment of Cancer (EORTC) QLQ-C30 and Euro-QOL 5 dimension 5 level version (EQ-5D-5L) in HLA-A*24:02-positive patients receiving S-588410.
- To collect data of tumor evaluation, PFS, OS, and EORTC QLQ-C30 and EQ-5D-5L questionnaires in HLA-A*24:02-negative patients without the study treatment.

3. INVESTIGATIONAL PLAN

3.1 Overall Study Design and Plan

The study is an open-label, multicenter study and consists of 2 study periods:

- The Screening Period is 28 days prior to enrollment. Potential patients who provided written informed consent will be screened for assessment of the eligibility in the screening period.
- The Treatment Period/Observation Period is 24 months (104 weeks) after enrollment. Eligible patients will be enrolled in the study, and assigned to S-588410 Group or Observation Group depending on the HLA-A genotype. Enrollment form will be reported via FAX.

**For Japanese study sites**	**Sponsor's study manager: [redacted], Clinical Research Department, Shionogi & Co., Ltd.**<br><br>**FAX: [redacted]**
**For EU study sites**	**Sponsor's medical monitor: [redacted] Medical Consultant Oncology, Shionogi Ltd.**<br><br>**Fax: [redacted]**

  - HLA-A*24:02-positive patients will be enrolled in the S-588410 Group. Each patient will receive 1 mL of S-588410 emulsion, which contains 1 mg each of S-288301 (DEPDC1-derived peptide), S-288302 (MPHOSPH1-derived peptide), S-488401 (URLC10-derived peptide), S-488402 (CDCA1-derived peptide), and S-488403 (KOC1-derived peptide). S-588410 will be injected subcutaneously in the patient's inguinal, axillary, or cervical region once weekly for 12 weeks and once every 2 weeks thereafter for up to 24 months. They will undergo efficacy assessments (specific CTL measurement, anti-tumor evaluation, PFS, OS, and EORTC QLQ-C30 and EQ-5D-5L questionnaire assessment), safety assessments (injection site assessment, physical examination, vital sign assessment, electrocardiogram [ECG], Eastern Cooperative Oncology Group Performance Status [ECOG PS], laboratory tests, and adverse event [AE] monitoring), and other assessments (antigen expression testing and T-cell receptor repertoire analysis).
  - HLA-A*24:02-negative patients will be enrolled in the Observation Group and will not receive the study drug. They will undergo the efficacy and safety assessments similarly to the S-588410 Group except for specific CTL measurement, and injection site assessment, and T-cell receptor repertoire analysis.

Follow-up assessments will be performed in all enrolled patients for 3 years after enrollment of the last patient. The following data will be reported to the sponsor: survival or death, date and site of progression, and date, description of, and response to subsequent therapy for the target disease (ie, bladder cancer).

The study design is shown in Figure 3-1. The study events table is shown in Appendix 1 and Appendix 2.

Figure 3-1 Study Schematic

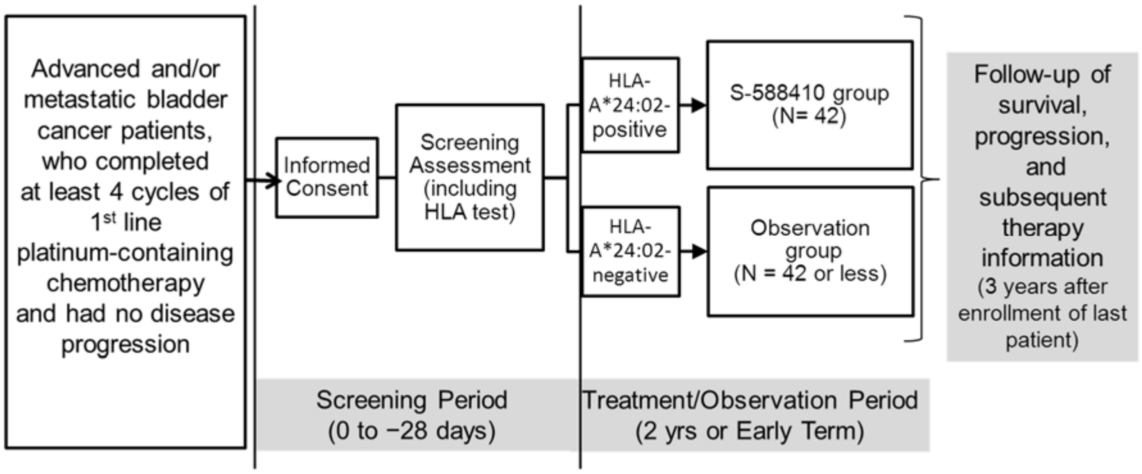

3.2 Rationale for Study Design

3.2.1 Rationale for the patient population

The patient population eligible for this study was selected based on the following reasons:

- As stated in the Food and Drug Administration (FDA) Guidance for Industry "Clinical considerations for therapeutic cancer vaccines October 2011" [14], patients with no evidence of residual diseases or minimal burden of disease may provide adequate time for the cancer vaccine to elicit a detectable immune response.
- Platinum-containing chemotherapy is recommended as the standard first-line chemotherapy in all regions of US and Europe, and Japan (GC or MVAC as the main choice) [3, 4, 5].
- For the majority of the patients, the disease will continue to progress even after completion of the first-line chemotherapy, as evidenced by the poor 5-year survival data.
- There are currently no treatments approved for maintenance therapy following first-line platinum containing chemotherapy in this patient population.
- Maintenance therapy (in patients after first-line chemotherapy including patients whose disease responds or remain stable) has been reported in several clinical studies [11, 12, 13].

3.2.2 Rationale for dosage of S-588410

The previous phase 1/2 studies of S-288310 (Study 0920P1611) and S-488410 (Study 1008P1711) suggested that there is no dose-relationship between the CTL induction and the tested doses (1 to 4 mg) and that the local symptoms at injection sites may be caused by adjuvant (Montanide ISA 51 VG). Since lower volume of subcutaneous injection is considered more tolerable for patients, 1 mg of S-588410 is selected as optimal dose for the

CTL induction.

As for duration of treatment, 24-months was selected as the appropriate duration since 24-months is approximately twice the duration of the reported median overall survival (10.3 months) [11] in bladder cancer patients after the first-line chemotherapy.

The dosing frequency was chosen based on the Study 0920P1611 and the Study 1008P1711 since the CTL induction within 12 weeks is already shown well at weekly injection and efficacy and safety are confirmed at weekly or every 2 weeks injection thereafter in the studies. Therefore the dosing frequency of S-588410 is chosen as weekly injection for 12 weeks, followed by every 2 weeks thereafter.

The method of administration is also selected based on the Study 0920P1611 and Study 1008P1711.

3.3 Study Duration

3.3.1 Study Duration in individual patients

The total study duration will be 108 weeks (Screening Period, 4 weeks; Treatment Period, 104 weeks).

3.3.2 Planned Study Duration for the Study

The planned study duration is from February 2014 to October 2018.

4. STUDY POPULATION SELECTION

4.1 Study Population

Male or female patients 20 years of age or older, with advanced and/or metastatic bladder cancer who satisfy the following eligibility criteria will be enrolled.

4.2 Inclusion Criteria

Patients who fulfill all of the following criteria assessed by the end of screening period will be included in the study.

1. Patients with advanced and/or metastatic bladder cancer (including urothelial cancer of renal pelvis, ureters, and urethra) who have CR, PR, or SD based on the Response Evaluation Criteria in Solid Tumors (RECIST) version 1.1 at the end of at least 4 cycles of first-line platinum-containing systemic chemotherapy.
   (Note: Patients who have subsequently received another platinum-containing chemotherapy due to intolerance* of first-line platinum-containing chemotherapy are eligible if the total number of cycles of platinum-containing regimens reached 4 cycles or more. The patients had no PD at the time of change of platinum-containing chemotherapy. *: any non-hematologic toxicity with grade 3 or higher, or hematologic toxicity with grade 4, according to Common Terminology Criteria for Adverse Events [CTCAE] version 4.03.)
2. Patients who are male or female aged ≥20 years at the time of informed consent.
3. Patients with the ECOG PS 0 or 1 at enrollment.
4. Patients who have a lymphocyte count accounting for 15% or higher of the total white blood cell count within 28 days before enrollment.
5. Patients who are willing and able to provide formalin-fixed and paraffin-embedded bladder cancer tissues for antigen expression measurement.
6. Patients who provide a personally signed and dated informed consent document for participation in the study.

4.3 Exclusion Criteria

Patients who meet any of the following criteria by the end of screening period will be excluded from the study.

1. Patients who have progressive disease (PD) on RECIST version 1.1 at the end of at least 4 cycles of first-line platinum-containing chemotherapy.
2. Patients who are judged to have clinically progressive symptoms at the end of at least 4 cycles of first-line platinum-containing chemotherapy by the investigator or subinvestigator.
3. (deleted)
4. Patients with a history of malignant cancer (except for carcinoma in situ or intra-mucosal cancer that resolved with endoscopic therapy) within 5 years before enrollment.
5. Patients who received any prior therapies for target disease within 3 weeks before the first administration of S-588410.
6. Patients who cannot receive the first administration of S-588410 within 12 weeks

after the last administration of chemotherapy.

7. Patients who are expected to require any of the following therapies between enrollment and completion or discontinuation of the study treatment.
   - Anti-malignant tumor drug
   - Systemic corticosteroid (except for corticosteroid defined as the equivalent of prednisone ≤ 10 mg/day orally)
   - Systemic immunosuppressant drug
   - Immunotherapy
   - Radiotherapy (except for restricted radiotherapy for pain relief of bone metastasis) for the target disease
   - Surgical therapy for the target disease
   - Hyperthermia for the target disease
   - Herbal medicine with anti-tumor or immunosuppressant effect
   - Other investigational new products
8. Patients who have severe (CTCAE version 4.03 grade 3 or higher) concurrent hepatic impairment, renal impairment, heart disease, hematological disease, respiratory disease, or metabolic disease, with the exception of any symptoms and/or signs associated with target disease.
9. Patients who have the following laboratory data with grade 3 or higher according to CTCAE version 4.03 criteria within 28 days before enrollment.
   - White blood cell count <2000/mm^3 or >100 000/mm^3
   - Platelet count <50 000/mm^3
   - Hemoglobin <8.0 g/dL
   - Aspartate aminotransferase (AST) or alanine aminotransferase (ALT) >5.0 × the upper limit of normal (ULN)
   - Total bilirubin >3.0 × ULN
   - Serum creatinine >3.0 × ULN
10. Patients who has an increase in an eosinophil counts (more than 5000/mm^3), within 28 days before enrollment, or patients with a history or current complication of eosinophilic pneumonia or interstitial pneumonia. If patients have symptoms and signs suggestive of pneumonia before enrolment, the investigator or subinvestigator must confirm the presence or absence of concurrent eosinophilic pneumonia or interstitial pneumonia on the basis of additional examinations such as chest x-ray.
11. Patients who have known human immunodeficiency virus infection.
12. Patients with uncontrolled systemic or active infection.
13. Patients who had any diseases with the risk of sudden death within 12 months before enrollment.
    [Examples]
    - Myocardial infarction
    - Unstable angina
    - Coronary or peripheral artery bypass graft surgery
    - Thrombotic or embolic events such as pulmonary embolism, deep vein thrombosis, or transient ischemic attack
14. Patients who have known brain metastases.
15. Patients with a history or evidence of autoimmune diseases and/or immunodeficiency disorders.

[Examples]
- Systemic lupus erythematosus
- Multiple sclerosis
- Systemic sclerosis
- Rheumatoid arthritis

16. Patients with a history of severe (CTCAE version 4.03 grade 3 or higher) allergic reaction to a drug, vaccination, or biological preparation.
17. Patients who are hospitalized for treatment at enrollment.
18. Female patients who are lactating or pregnant. Female patients who are of childbearing potential, not included in any of the following, are positive for the pregnancy test at enrollment.
    - Postmenopausal woman (at least 2 years since their last regular menstrual period without any other medical reason)
    - Women who are surgically sterile by hysterectomy and/or bilateral oophorectomy, or by tubal ligation
19. Patients who cannot or do not intend to practice effective contraception (barrier contraceptives [male condom, female condom, or diaphragm with a spermicidal gel]; hormonal contraceptives [implants, injectables, combination oral contraceptives, or transdermal patches]; or intrauterine devices) as per physicians' recommendations from enrollment to 90 days after completion or discontinuation of study treatment.
20. Patients who received DEPDC1, MPHOSPH1, URLC10, CDCA1, or KOC1 peptide vaccines before.
21. Patients who received any other investigational products within 28 days or 5 half-lives of the investigational products before enrollment whichever is longer.
22. Patients who are considered ineligible for this study by the investigator or subinvestigator due to any reasons, including inability to understand and follow the requirements of the study.

5. STUDY TREATMENT(S)

5.1 Description of Treatment(s)

5.1.1 Test Drug

The study drug (S-588410) is a freeze-dried injectable formulation that contains 2.4 mg of each active ingredients of the following 5 peptides, S-288301 (a DEPDC1-derived peptide), S-288302 (a MPHOSPH1-derived peptide), S-488401 (a URLC10-derived peptide), S-488402 (a CDCA1-derived peptide), and S-488403 (a KOC1-derived peptide), and the inactive excipients sucrose, sodium hydroxide, and hydrochloric acid in a vial. The vials contain quantities of S-588410 that are higher than the administration dose to compensate for assumed loss upon preparation. S-588410 is manufactured by Shionogi & Co., Ltd. (Osaka, Japan).

5.1.2 Adjuvant

An adjuvant contains 1.2 mL of Montanide ISA 51 VG, which is defined as a mixture of a highly purified mineral oil and a surfactant (mannide monooleate), in a vial. The adjuvant is manufactured by Shionogi & Co., Ltd. (Osaka, Japan).

5.2 Treatments to be Administered

5.2.1 Method for Preparation

S-588410 is reconstituted with 1.2 mL of water for injection, then mixed and emulsified with equal amount of the adjuvant just before administration. A connector device is used to connect the 2 syringes (one with reconstituted S-588410 solution and the other with adjuvant) for the mixing step required to form the emulsion. The mixing step is then performed by pushing the 2 plungers of both syringes ten times or more (five times or more per one syringe) reciprocally. Details on the method of preparing the emulsion and storing the emulsion will be stipulated in the separate following manual: S-588410 Study Drug Preparation Manual.

5.2.2 Treatments to be Administered

Patients will subcutaneously receive 1 mL of S-588410 emulsion, which includes 1 mg each of the 5 peptides, once weekly for 12 weeks and once every 2 weeks thereafter for up to 24 months. The study drug will be injected subcutaneously in the patient's subaxillary, inguinal, or cervical region. In case subcutaneous injection in the above regions is difficult due to reactions at the injection site, the study drug will be injected in the proximity of the above region to the extent possible.

5.3 Selection and Timing of Dose for Each Patient

In the S-588410 Group, each patient will receive S-588410 in the same dosage at the specified time points.

5.4 Method of Assigning Patients to Treatment Groups

Eligible patients will be enrolled in the study, and assigned to the S-588410 Group or the Observation Group depending on the HLA-A genotype. In the S-588410 Group, each

patient will receive S-588410 in the same dosage at the specified time points. In the Observation Group, each patient will not receive any study drug.

5.5 Blinding

This is an open-label study and blinding will not be performed in the study.

5.6 Management of Clinical Supplies

5.6.1 Study Drug Packaging and Storage

The sponsor will provide adequate supplies of S-588410 and adjuvant for distribution to the study centers. The S-588410 box will contain 4 vials of S-588410. The boxes will be labeled with the code of investigational product (S-588410), the name of active ingredient, dosage form, packaged (batch) number, month of manufacture or expiry, storage conditions, caution statements for clinical study use only, name of sponsor, and address of sponsor. The adjuvant box will contain 4 vials of adjuvant. The boxes will be labeled with the identification name (Montanide ISA 51 VG), dosage form, volume, packaged (batch) number, month of manufacture or expiry date, storage conditions, caution statements for clinical study use only, name of sponsor, and address of sponsor.

S-588410 and the adjuvant must be stored in a secure area under the appropriate physical conditions described on the label for the product(s).

5.6.2 Study Drug Accountability

The sponsor will supply the study drug to the person responsible for study drug handling, which is a designee of the head of the medical institution, under the study contract between the sponsor and the medical institution. The person responsible for study drug handling will handle the study drug according to procedures for method of storage and drug accountability record as specified in a separate document. The expired drug can be returned to the sponsor during the study.

All unused drug supplies will be held in the medical institution although those supplies will not be required to be stored under the storage conditions defined above. After the person responsible for study drug handling records accurate amounts of unused drug supplies at the completion of the study, the unused drug supplies in appropriate boxes will be returned to the sponsor with a copy of overall drug accountability record.

5.7 Other Supplies

The syringes and the mixing connector (refer to Section 3.2 in the current IB for S-588410) used to connect the 2 syringes (one with reconstituted S-588410 solution and the other with adjuvant) for the preparation of the emulsion will be provided by the sponsor.

6. RESTRICTIONS

6.1 Prior Therapy

Prior therapies are defined as therapies which were taken prior to enrollment in the study.

6.1.1 Prior Therapy for the Target Disease

The investigator or subinvestigator will record the information listed below on any therapy for the target disease in the case report form (CRF). The first-line chemotherapy for the target disease includes intra-arterial infusion chemotherapy and intravesical infusion chemotherapy (neo-adjuvant chemotherapy before radical cystectomy and adjuvant chemotherapy after radical cystectomy are excluded from the first-line chemotherapy).

- Name of used drug or used procedures (including surgical procedures)
- Dose change, as-needed use, and route of administration (if a drug is administered)
- Duration of treatment (start and stop date)
- Number of cycles (if chemotherapy)

The investigator or subinvestigator will record the best overall response for target disease at prior therapy and the objective tumor response for the target disease at the end of prior therapy with the exception of surgical procedures based on RECIST version 1.1 in the CRF. Also, the investigator or subinvestigator will submit all imaging reports through the start and the end of first-line chemotherapy to the sponsor.

6.1.2 Prior Therapy for Other Than the Target Disease

The investigator or subinvestigator will record the information listed below on any therapy (prescription drugs, over-the-counter drugs, procedures without any medication) for other than the target disease taken by patient within 2 weeks prior to enrollment in the study in the CRF.

- Name of used drug or used procedures
- Dose change, as-needed use, and route of administration (if a drug is administered)
- Duration of treatment (start and stop date)
- Reason for use

6.2 Concomitant Therapy during the Study

Concomitant therapies are defined as therapies for Treatment/Observation Period.

The investigator or subinvestigator will record the following information for all therapies (prescription drugs, over-the-counter drugs, procedures without any medication) used or the Treatment/Observation Period in the CRF.

- Name of used drug or used procedures
- Dose change, as-needed use, and route of administration (if a drug is administered)
- Duration of treatment (start and stop date)
- Reason for use

6.2.1 Prohibited Therapy

The implementation of the following therapies is not permitted during the Treatment/Observation Period.

- Anti-malignant tumor drug
- Systemic corticosteroid (except for corticosteroid defined as the equivalent of prednisone ≤ 10 mg/day orally)
- Systemic immunosuppressant drug
- Immunotherapy
- Radiotherapy (except for palliative radiotherapy for bone metastasis) for the target disease
- Surgical therapy for the target disease
- Thermotherapy for the target disease
- Herbal medicine with anti-tumor or immunosuppressant effect
- Any other investigational new products

6.3 Other Provisions

In the S-588410 Group, patients will rest for at least 30 minutes after the initial dose, and will be closely monitored under a physician's supervision.

7. STUDY PROCEDURES AND METHODS OF ASSESSMENTS

7.1 Informed Consent

The investigator or subinvestigator will fully explain the nature of the study to a patient using the institutional review board (IRB)/institutional ethics committee (IEC)-approved informed consent document. When the patient agrees to participate in the study, the patient must voluntarily sign a consent form prior to the initiation of any study procedures. A copy of the signed and dated informed consent document will be given to the patient. The signed and dated original consent form will be retained by the investigator. Informed consent will be obtained from all patients. A patient cannot be entered the study until he/she has signed and dated on the consent form.

The investigator or subinvestigator is responsible for ensuring that the patient understands the risks and benefits of participating in the study, including answering any questions the patient may have throughout the study and sharing any new information in a timely manner that may be relevant to the patient's willingness to continue his/her participation in the study.

7.2 Baseline Information

7.2.1 Baseline Patient Characteristic and Medical History

The following baseline patient characteristics and medical history will be obtained at the initiation of the study and entered in the CRF:

- Baseline patient characteristics
  - Date of enrollment, date of patient's written informed consent, date of birth, sex, ethnicity, and race
  - TNM Clinical Classification (T, primary tumor; N, regional lymph nodes; M, distant metastasis)[*1] at enrollment in the study
- Medical history
  - Information related to the target disease[*1] (at the time of definitive diagnosis): Primary lesion, date of definitive diagnosis, TNM Clinical Classification[*1], histology, G histopathological grading
  - Surgical history for the target disease (see Section 6.1.1)
  - Previous disease other than the target disease (cancer or conditions that required hospitalization within 1 year before enrollment), and complication (with exception of any symptoms and/or signs associated with target disease)
  - Prior therapy for the target disease and other than the target disease (see Section 6.1)

*1: In accordance with TNM Classification of Malignant Tumours 7th Edition by UICC (Union for International Cancer Control).

7.2.2 Human Leukocyte Antigen-A Gene Test

Patients for whom the HLA-A type has not yet been identified will be assessed for HLA-A genotypes with four-digit alleles. A blood sample (2 mL) for HLA-A genotyping should be collected in whole blood collection tubes at screening. Blood samples should be stored at temperature between 1°C and 10°C.

The HLA-A genotyping should be performed at the central laboratory (see also Section 9.1) or the laboratory divisions (or their contract laboratory) in the study sites.

Results of HLA-A genotypes with four-digit alleles will be entered in the CRF.

7.3 Efficacy Assessments

7.3.1 Specific Cytotoxic T Lymphocyte Measurement

In the S-588410 Group, a blood sample (40 mL) for measurement of CTL induction will be collected in blood collection tubes containing EDTA-2Na throughout the study at the time points specified in the schedule of events (Appendix 1). Prior to shipment by the delivery company (see also section 9.1), samples should be stored at room temperature (between 1°C and 30°C).

Isolation of the peripheral blood mononuclear cells (PBMCs) should begin at the central laboratory (see also section 9.1) within 24 hours of the sample collection. At the same time, 4 mL of plasma will be extracted from the blood sample for CTL measurement for possible exploratory analysis of biomarkers (not including gene analysis).

Isolated PBMCs will be stored in the vapor phase of liquid nitrogen until shipment to the laboratory for specific CTL measurement (see also section 9.1). These shipments should be done using dry liquid nitrogen shippers.

CTL activity will be measured by using an Enzyme-Linked Immunosorbent Spot (ELISPOT) method to detect the production of interferon-γ at the laboratory for specific CTL measurement in accordance with the laboratory instruction manual. The results of the CTL activity will be provided based on interferon-γ production quantity with the following 4 categories: (−), no specific CTL activity; (+), specific CTL activity; (2+), strong specific CTL activity; and (3+), extremely strong specific CTL activity.

7.3.2 Tumor Evaluation

In both the S-588410 and the Observation Groups, a computed tomography (CT) scan or an magnetic resonance imaging (MRI) scan will be performed in accordance with a separate imaging manual throughout the study at the time points specified in the schedule of events (Appendix 1 and Appendix 2). All original images or those copies will be submitted to the central imaging for central review (see also section 9.1).

Tumor evaluation, based on RECIST Version 1.1 and immune-related response criteria (irRC), will be performed by central review with the following categories: CR, PR, SD, and PD for the RECIST; irCR, irPR, irSD, and irPD for the irRC. A status of stable disease will only be assigned if the evaluation is unchanged for at least 6 weeks after enrollment. A status of CR or PR will only be assigned if the evaluation is unchanged for at least 4 weeks.

Tumor evaluation will also be assessed for any response in image analysis such as tumor cavitation based on the criteria stipulated in the separated manual by central review (see also section 9.1).

7.3.3 Evaluation of Progression-Free Survival and Overall Survival

For all enrolled patients, the investigator or subinvestigator will investigate the following items during the Treatment/Observation Period, and then also examine those items every 6 months for up to 3 years after the date the last patient is enrolled. The investigator or subinvestigator will record the results in the Vital Status Follow-up Form (see Appendix 6) and submit to the sponsor.

- Survival or death, and date of determination (date of death if patient has died), as well as the cause of death and autopsy findings if the patient has died
- Progression or no progression of target disease, and date of determination (date and site of progression if progressed)

The information listed below on any subsequent therapy for the target disease will also be recorded in the Vital Status Follow-up Form (see Appendix 6) and will be submitted to the sponsor.

<Therapy other than surgical therapy>

- Name of used drug or used procedures
- Dose and number of cycles (if chemotherapy)
- Duration of treatment (start and stop date)
- Best overall response and response at the end of therapy based on RECIST version 1.1
- Reason for stopping therapy

<Surgical therapy>

- Resection site
- Date of surgical therapy

7.3.4 QOL Assessment

In both the S-588410 and the Observation Groups, the general health status of patients will be assessed using the EORTC QLQ-C30 (see Appendix 4) and EQ-5D-5L questionnaires (see Appendix 5) throughout the study at the time points specified in the schedule of events (Appendix 1 and Appendix 2).

The EORTC QLQ-C30 is an integrated system for assessing the health-related quality-of-life of cancer patients participating in international clinical trials. The EORTC QLQ-C30 is composed of multi-item scales and single-item measures, results in 30-item questionnaires. This includes a global health status/quality-of-life scale, five functional scales (physical, role, emotional, cognitive, and social), three symptom scales (fatigue, nausea and vomiting, and pain), and six single items assessing additional symptoms commonly reported by cancer patients (dyspnea, insomnia, loss of appetite, constipation, and diarrhea) and perceived financial impact of the disease. The raw QLQ-C30 scores will be recorded in the CRF, and transformed to scores ranging from 0 to 100 for analysis according to the EORTC QLQ-C30 Scoring Manual.

EQ-5D is a standardized measure of health status developed by the EuroQol Group in order to provide a simple, generic measure of health for clinical and economic appraisal.

The EQ-5D-5L consists of 2 pages - the EQ-5D-5L descriptive system and the EQ visual analog scale (EQ VAS). The descriptive system comprises the following 5 dimensions: mobility, self-care, usual activities, pain/discomfort, and anxiety/depression. Each dimension has 5 levels: no problems, slight problems, moderate problems, severe problems, and extreme problems. The raw EQ-5D-5L score, defined by the EQ-5D-5L descriptive system, will be recorded in the CRF, and converted into a single index value.

The EQ VAS records the subject's self-reported current health-status on a 20 cm vertical, visual analogue scale with endpoints anchored on 100 = best imaginable health and 0 = worst imaginable health. The self-reported EQ VAS score will be recorded in the CRF.

7.4 Safety Assessments

7.4.1 Assessment of Injection Site

In the S-588410 Group, an injection site reaction will be assessed for signs (ie, itching, erythema, induration, and ulcer) just before injection between Visit 2 and Visit 14. Results of assessment will be recorded in the CRF. An injection site reaction will also be recorded as AE in the CRF.

7.4.2 Physical Examination

In both the S-588410 and the Observation Groups, a physical examination will be performed throughout the study at the time points specified in the schedule of events (Appendix 1 and Appendix 2). Weight in kilograms will be obtained at screening and Visit 59 (or Discontinuation). Height in centimeters will be obtained at screening. Weight and height will be entered in the CRF.

7.4.3 Performance Status

In both the S-588410 and Observation Groups, the investigator will evaluate ECOG PS grade (Table 7-1) throughout the study at the time points specified in the schedule of events (Appendix 1 and Appendix 2). Results of evaluation will be entered in the CRF.

Table 7-1 ECOG PS

Grade	Performance status[a]
0	Fully active, able to carry on all pre-disease performance without restriction
1	Restricted in physically strenuous activity but ambulatory and able to carry out work of a light or sedentary nature, eg, light house work, office work.
2	Ambulatory and capable of all selfcare but unable to carry out any work activities. Up and about more than 50% of waking hours
3	Capable of only limited selfcare, confined to bed or chair more than 50% of waking hours.
4	Completely disabled. Cannot carry on any selfcare. Totally confined to bed or chair.

[a] Based on the response criteria of the Eastern Cooperative Oncology Group [15].

7.4.4 Vital Signs

In both the S-588410 and Observation Groups, blood pressures (systolic / diastolic pressures), body temperature, and pulse rate will be measured throughout the study at the time points specified in the schedule of events (Appendix 1 and Appendix 2).

Blood pressures and pulse rate will be measured after the patient has been in a sitting position for at least 3 minutes.

The investigator or subinvestigator will consider whether any abnormal changes from baseline (Day 1) are clinically significant (see also Section 7.6.7). Clinically significant changes will be reported as an AE on the CRF. Results of blood pressures, body temperature, and pulse rate will be entered in the CRF.

7.4.5 Electrocardiogram

In both the S-588410 and the Observation Groups, a standard ECG will be performed throughout the study at the time points specified in the schedule of events (Appendix 1 and Appendix 2) after resting in a supine or semi-recumbent position for at least 3 minutes.

The investigator or subinvestigator will assess whether the ECG is normal or abnormal. If the ECG is abnormal, the investigator or subinvestigator will consider further whether the abnormal ECG is clinically significant (see also Section 7.6.7). Clinically significant changes will be reported as an AE on the CRF. Result of ECG and its interpretation will be entered in the CRF.

7.4.6 Clinical Laboratory Tests

7.4.6.1 Routine Laboratory Tests

In both the S-588410 and the Observation Groups, blood and urine samples for clinical laboratory assessments will be collected throughout the study at the time points specified in the schedule of events (Appendix 1 and Appendix 2). Routine laboratory tests, as presented in Table 7-2, will be performed.

Table 7-2 Routine Laboratory Tests

Category	Evaluation items
Hematology tests	hematocrit, hemoglobin, platelet count, red blood cell count, and white blood cell count with differential (neutrophils, basophils, lymphocytes, eosinophils, monocytes)
Blood chemistry tests	alkaline phosphatase (ALP), ALT, AST, total bilirubin, gamma glutamyl transferase (GGT), lactate dehydrogenase (LDH),<br>blood urea nitrogen (BUN), creatinine,<br>chloride (Cl), calcium (Ca), potassium (K), sodium (Na),<br>total protein, albumin, uric acid, creatine kinase (CK),<br>total cholesterol, triglycerides,<br>and C reactive protein (CRP)
Urinalysis	glucose, occult blood, protein, and urobilinogen

Routine laboratory (blood chemistry, hematology, others, and urinalysis) test samples will be analyzed at the central laboratory (see also Section 9.1).

The investigator or subinvestigator will assess whether any abnormal changes from baseline (screening) are clinically significant (see also Section 7.6.7). Clinically significant changes will be reported as an AE on the CRF.

7.4.6.2 Pregnancy Tests

Pregnancy test will be performed only for female patients with the exception of patients who are postmenopausal (at least 2 years since their last regular menstrual period without any other medical reason) or are surgically sterile by hysterectomy and/or bilateral oophorectomy, or by tubal ligation. Urine or serum pregnancy test will be performed at screening in both the S-588410 and the Observation Groups, and Visit 59 (or Discontinuation) in the S-588410 Group.

7.4.6.3 Immunological Tests

The viral test for human immunodeficiency virus, hepatitis B surface antigen, and hepatitis C virus antibody will be performed at screening. Test samples will be analyzed at the central laboratory.

7.5 Other Assessments

7.5.1 Antigen Expression Testing

Patients will provide archival tumor tissue (ideally 5 mm × 5 mm × 0.5 mm in size) resected by surgery or biopsy at screening in order to measure antigen expression levels for DEPDC1, MPHOSPH1, URLC10, CDCA1, KOC1, Programmed death-ligand 1 (PD-L1), and HLA-Class I etc. by means of immunohistochemistry. If archival tissue is not available, biopsies must be performed at screening to obtain sufficient amounts of tumor tissue for measurement of antigen expression levels. Optional biopsies will be performed at discontinuation in order to measure these antigen expression levels following study drug administration (if possible).

Prior to shipment by the delivery company (see also section 9.1), tumor tissue samples should be stored at room temperature (between 1°C and 30°C). Tumor tissue samples should be transported to the laboratory for antigen expression testing (see also section 9.1) by the delivery company, and analyzed at the laboratory.

7.5.2 T-cell receptor Repertoire Analysis

In the S-588410 Group, a blood sample (10 mL) for the T-cell receptor (TCR) repertoire analysis will be collected in blood collection tubes containing EDTA-2Na throughout the study at the time points specified in the schedule of events (Appendix 1). Prior to shipment by the delivery company (see also section 9.1), samples should be stored at room temperature (between 1°C and 30°C). Isolation of the PBMCs should begin at the central laboratory (see also section 9.1) within 24 hours of the sample collection. Isolated PBMCs will be stored in the vapor phase of liquid nitrogen until shipment to the laboratory for preparation of complementary DNA (cDNA) (see also section 9.1). These shipments should be done using dry liquid nitrogen shippers. Messenger RNA (mRNA) will be extracted from the PBMCs, and then complementary DNA (cDNA) will be synthesized at the laboratory for preparation of cDNA. Synthesized cDNA will be transported to the laboratory for TCR repertoire analysis (see also section 9.1) by the delivery company.

The cDNA will be used for a newly established method for monitoring immune responses to S-588410. The TCR repertoire analysis is a method that obtains the information of millions of TCR alpha and beta chains and identifies possible TCRs, which recognize a HLA peptide complex, in CTLs responding to S-588410, in a high sensitive and quantitative way. The cDNA will be used for searching/identifying such TCR clones. The study for the TCR repertoire analysis is conducted by [redacted] (see also section 9.1) and details of the study plan are specified in the protocol by [redacted]. All results in the analysis will be kept confidential and, if presented, all data will be displayed with unidentified subject information. No information that may identify each subject should be disclosed. Results of TCR analysis will be reported in separate documents from the main study results.

The sample will be given a specific number to protect personal information when it is collected. The sponsor will not disclose subject personal information to any other institutions (eg, the central laboratories and the delivery companies). The sponsor may

ask to store the sample in the storage company (see section 9.1) for up to 10 years after the last subject ie enrolled in the study or the sponsor may discard the sample(s) at an earlier time. Subjects can request excluding his/her sample from testing or discarding PBMCs, mRNA, and cDNA obtained from the blood sample anytime; however, TCR analysis results can be not deleted once the testing is performed.

7.6 Adverse Events Assessments

7.6.1 Performing Adverse Events Assessments

AEs will be found by the patient's spontaneous complaint, or as a result of nonleading questions, physical examination, vital signs, or laboratory tests. AEs include any occurrences that are new in onset or aggravated in severity or frequency from the baseline condition, or abnormal results of diagnostic procedures, including laboratory test abnormalities. Medical histories which are reported at the baseline and worsen will be considered as AEs. Aggravation of target disease (eg, tumor growth and the appearance of new lesion) or any symptoms and/or signs associated with target disease (eg, cancer pain and carcinomatous pleural effusion) result in any of the outcomes such as death or life-threatening condition in Section 7.6.8.1, they must be considered as AEs and serious AEs (SAEs). Aggravations of target disease other than those above are not considered as AEs in the study because they will be evaluated as other assessments (eg, Tumor evaluation, QOL assessment, and ECOG [PS]).

The investigator or subinvestigator will assess AEs in the S-588410 Group and the Observation Group. AEs should be fully investigated and recorded in detail including onset date, date of outcome assessment (if outcome is other than not recovered or unknown), severity, seriousness with a category of seriousness, relationship with the study drug, action taken to manage the AE, and outcome of the AE in the CRF.

7.6.2 Timing

AEs will be collected from the time of informed consent through Visit 59 (or Discontinuation). Any SAE in the S-588410 Group assessed as causally related to the study drug and ongoing at Visit 59 (or Discontinuation) will be followed until resolution, stabilization, the condition becomes chronic, or the patient is lost to follow-up. Any AEs ongoing at Visit 59 (or Discontinuation) will be followed until resolution, stabilization, the condition becomes chronic, or 30 days after Visit 59 (or Discontinuation).

7.6.3 Severity

The severity of an event will be graded by the investigator or subinvestigator according to CTCAE version 4.03.

Grade 1	Mild; asymptomatic or mild symptoms; clinical or diagnostic observations only; intervention not indicated
Grade 2	Moderate; minimal, local or noninvasive intervention indicated; limiting age-appropriate instrumental activities of daily living[a]
Grade 3	Severe or medically significant but not immediately life threatening; hospitalization or prolongation of hospitalization indicated; disabling; limiting self-care activities of daily living[b]
Grade 4	Life-threatening consequences; urgent intervention indicated
Grade 5	Death related to AE

A semicolon indicates "or" within the description of the grade.

[a] Instrumental activities of daily living refer to preparing meals, shopping for groceries or clothes, using the telephone, managing money, etc.

[b] Self-care activities of daily living refer to bathing, dressing and undressing, feeding self, using the toilet, taking medications, and not bedridden.

7.6.4 Relationship

The relationship of an event to the study drug will be determined by the investigator or subinvestigator according to the following criteria:

- Related : An AE which can be reasonably explained that the study drug caused the AE. For example, the occurrence of the AE cannot be explained by other causative factors, can be explained by pharmacological effect of the study drug such as similar event had been reported previously, or increase/decrease of the dose affected the occurrence or seriousness of the AE, etc.
- Not related: An AE which cannot be reasonably explained that the study drug caused the AE.

Adverse drug reactions (ADRs) are defined as any drug-related treatment-emergent AE (TEAE) of which relationship is considered to be related.

7.6.5 Expectedness

An expected ADR is any ADR that is consistent with the current IB for S-588410. As stated in the guidance of IB, if patients receiving S-588410 start having symptoms and signs which suspect interstitial pneumonia, additional tests (eg, KL-6, Chest CT) will be performed. When there are any signs or symptoms of pneumonia, SpO_2 (arterial oxygen saturation) will be measured if needed. When eosinophil counts are more than $5000/mm^3$ [16], additional tests (eg, Chest X ray) will be performed, even though there are no signs or symptoms of pneumonia.

7.6.6 Significant Adverse Events

Any AE that leads to withdrawal of study treatment or is grade 3 or higher in the S-588410 Group will be assessed.

7.6.7 Clinical Laboratory Adverse Events

For any abnormal laboratory test results (hematology, blood chemistry, or urinalysis) or other safety assessments (eg, physical examination, vital signs) that are worsening from baseline, the investigator or subinvestigator will consider whether their results are clinically significant. Abnormal laboratory test results are defined as values outside the reference range. For test results which are abnormal at baseline and significantly worsen following the initiation of the study, the investigator or subinvestigator must also consider whether their results are clinically significant. Any test results which are considered to be clinically significant by the investigator or subinvestigator are to be recorded as AEs. If abnormal laboratory finding is associated with disease or organ toxicity, the investigator should report only the disease or organ toxicity.

The investigator or subinvestigator will consider that those test results are clinically significant in the following circumstances:

- Test result leads to any of the outcomes included in the definition of an SAE.
- Test result leads to a discontinuation from the study.
- Test result leads to a concomitant drug treatment or other therapy.
- Test result requiring additional diagnostic testing or other medical intervention.
- Test result meeting the management and discontinuation criteria for liver function abnormalities identified in the Appendix 3.

In other circumstances than those as shown above, the investigator or subinvestigator will consider clinical significance at their own discretion.

In addition, when any test result met the management and discontinuation criteria for liver function abnormalities (Appendix 3), the results of further assessments and required follow-up should be recorded in the Liver Event Form.

7.6.8 Serious Adverse Events

7.6.8.1 Definition

An SAE is defined by regulation as any AE occurring at any dose that results in any of the following outcomes:

- death
- life-threatening condition
- hospitalization or prolongation of existing hospitalization
- persistent or significant disability/incapacity
- congenital anomaly/birth defect
- other medically important condition.

Important medical events that may not result in death, be life threatening, or require hospitalization may be considered as SAE when, based upon appropriate medical judgment, they may jeopardize the patient and may require medical intervention to prevent one of the outcomes listed in this definition. Examples of such medical events include allergic bronchospasm requiring intensive treatment in an emergency room or at home, blood dyscrasias or convulsions that do not result in inpatient hospitalization, or the development of drug dependency or drug abuse. The investigator or subinvestigator will determine the seriousness of AEs.

For the following criteria, termed "Hy's law", shown in the management and discontinuation criteria for liver function abnormalities (Appendix 3), the case must be reported as an SAE.

- AST or ALT >3 × ULN and total bilirubin >2 × ULN or PT-INR >1.5

Aggravation of target disease (eg, tumor growth and the appearance of new lesion) or any symptoms and/or signs associated with target disease (eg, cancer pain and carcinomatous pleural effusion) result in any of the outcomes such as death or life-threatening condition, they must be reported as SAEs.

7.6.8.2 Reporting Serious Adverse Events

All SAEs defined in 7.6.8.1 must be reported regardless of causal relationship to the study drug to the sponsor within 24 hours from the point in time when the investigator first becomes aware of the SAE. However, SAEs in the Observation Group are permitted to be reported to the sponsor within 30 days from the point in time when the investigator first becomes aware of the SAE. The investigators in Japanese study sites must complete the SAE form in detail to report to the sponsor. The investigators in EU study sites must complete the appropriate pages of the CRF with information relevant to the event and notify [redacted] by faxing the SAE Notification form. When the CRF is not available, the investigators may report to [redacted] in detail on the SAE form. A sample of the SAE form can be found in the Site Regulatory Binder. Follow-up information on the SAE may be requested by the sponsor.

When reporting SAEs, the investigator should record the diagnosis whenever possible. If

no diagnosis is available at the time of reporting, individual signs and symptoms can be reported.

**For Japanese study sites**	**Sponsor's study manager: [redacted],**<br>**Clinical Research Department, Shionogi & Co., Ltd.**<br>**TEL: [redacted]**<br>**FAX: [redacted]**
**For EU study sites**	[redacted]<br>**Tel: [redacted]**<br>**Fax: [redacted]**<br>**E-mail: [redacted]**

If the sponsor requires the follow-up assessment, the investigator should provide new information to the sponsor as it becomes available using the SAE Form and then faxed to the number listed above. Discharge summaries, consultant reports, autopsy reports, or other relevant documents must be evaluated by the investigator and all relevant information must be included on the follow-up SAE form. Copies of these reports may also be requested by the sponsor.

Appropriate remedial measures should be taken by the investigator using his/her best medical judgment to treat the SAE. These measures and the patient's response to these measures should be recorded. Clinical, laboratory, and diagnostic measures should be employed by the investigator as needed to adequately determine the etiology of the event.

After AE assessment specified in section 7.6.2, any SAEs which is considered to be related to study drug by the investigator must be reported to the sponsor.

All SAEs will be reported to Country Regulatory Authorities, IRB, or IEC as per local legal requirements.

7.6.9 Special Situations-Abuse, Misuse, Overdose, and Medication Error

Abuse, misuse, overdose, or medication error (as defined below) of S-588410 must be reported to the sponsor via fax by the investigator using a Special Situations Report Form as soon as possible (refer to 7.6.8.2, for reporting destination). If there are associated SAE, investigator will complete SAE Form as well.

- Abuse - persistent or sporadic, intentional excessive use of an investigational product(s), which is accompanied by harmful physical or psychological effects.
- Misuse - Intentional and inappropriate use of an investigational product(s) other than as directed or indicated at any dose. Cases of patients missing visits and also doses of investigational product(s) are not considered reportable as misuse.
- Overdose - Intentional or unintentional intake of a dose of investigational product(s) higher than the determined dose in the study protocol.
- Medication Error - any unintended error in the preparing or administration of an investigational product(s). Medication errors are reportable only as defined below. Cases of patients missing visits and also doses of investigational product(s) are not considered reportable as medication error.
  - The administration or consumption of the undetermined treatment is always reportable as a medication error.
  - Administration of an expired product should be considered as a reportable medication error.

7.6.10 Pregnancy

If a female patient in the S-588410 Group becomes pregnant during the study, she must be instructed to discontinue all study drugs and inform the investigator immediately. All pregnancies that occur after the first dose of the study drug through the follow-up period must be reported within 24 hours of becoming aware of the pregnancy and the Pregnancy Form be faxed to the sponsor by the investigator (refer to 7.6.8.2, for reporting destination). Pregnancy complications and elective terminations for medical reasons must be reported as an AE or SAE as appropriate. Spontaneous abortions must be reported as an SAE. The outcome of the pregnancy should be followed by the study site and must be reported using the Pregnancy Form, which must be faxed to the sponsor. All pregnancies that are confirmed after the follow-up visit but within 6 weeks of last study drug dose should be reported to the sponsor. At the end of the pregnancy (ie, birth, miscarriage, abortion) for the patient, the outcome should be reported to the sponsor.

In addition, the investigator (or subinvestigator) must attempt to collect pregnancy information on any female partners of male patients in the S-588410 Group who become pregnant while the patient is enrolled in the study. Pregnancy information must be reported to the sponsor as described above.

7.6.11 Treatment-Emergent Adverse Events

AEs reported after the initial dose of study drug in the S-588410 Group will be considered TEAE.

7.7 Withdrawal of Patients from the Study or Study Drug

The investigator or subinvestigator will make every reasonable attempt to complete the study. A patient may withdraw for any reason. The investigator or subinvestigator will advise the sponsor about the withdrawal of any patient.

The investigator will withdraw a patient from the study for any of the following reasons:

- When the investigator or subinvestigator determines that the patient is required to be initiated chemotherapy due to aggravation of the target disease.
- When the patient's PS (ECOG) becomes 3 or higher.
- When eosinophil counts are more than 5000/mm^3
- When a definitive diagnosis of eosinophilic pneumonia or interstitial pneumonia is made and it is considered to be related to the study drug
- When the patient requests to be withdrawn from the study.
- When the patient is proved to be ineligible for the study after participating in the study.
- When the patient is lost to follow-up.
- When the patient should be withdrawn from the study based on the management and discontinuation criteria for liver function abnormalities (Appendix 3).
- When the investigator or subinvestigator determines that the patient should be withdrawn because of other reasons.

In the event of a patient's withdrawal, the investigator will promptly notify the sponsor and will make every effort to complete the early termination assessments. All patients withdrawn due to AEs will be followed until resolution of any AEs until the unresolved AEs are judged by the investigator to have stabilized, or the patient is lost to follow-up.

7.8 Appropriateness of Measurements

High CTL induction rate was shown in the previous phase 1/2 studies of S-288310 (Study 0920P1611) and S-488410 (Study 1008P1711). Almost all vaccinated patients revealed CTL induction within 12 weeks after initial dose. The CTL induction rate within 12 weeks after initial dose will be selected as the primary endpoint in this study.

As stated in the FDA Guidance for Industry "Clinical Considerations for Therapeutic Cancer Vaccines", important clinical outcomes (eg, increased survival, symptomatic improvement) should be included as endpoints to assess clinical benefits and are required to support approval in a late phase trial [14]. The FDA Guidance for Industry "Clinical Trial Endpoints for the Approval of Cancer Drugs and Biologics" states that survival is considered the most reliable cancer endpoint and the preferred endpoint when studies can be conducted to adequately assess survival and survival improvement should be analyzed as a risk-benefit analysis to assess clinical benefit [17]. "Guideline for Clinical Evaluation of Anti-Malignant Tumour Agents", issued by Ministry of Health, Labour and Welfare in Japan, also states that the survival rate, the survival time, and survival information should be selected as the primary endpoint in a phase 3 study [18]. The European Medicines Agency guideline "Guideline on the evaluation of anticancer medicinal products in man" also states that convincingly demonstrated favourable effects

on survival are the most persuasive outcome of a clinical trial [19]. According to these guidelines, overall survival will be selected as the second endpoint in this study.

The other efficacy measures selected for the study are commonly used in clinical trials of cancer patients.

The safety evaluations selected for the study are typical of those for this patient population and type of investigation, and utilize widely accepted measures.

7.9 Acceptable Time Window

All assessments will be performed according to the schedule as shown in Appendix 1 and Appendix 2. The following acceptable time window, as shown in Table 7-3, can be accepted even if the timing of the measurement departs from the schedule specified in Appendix 1 and Appendix 2. Data departed from this time window will be handled as missing data for the study visit. However, skipping of Visit may be allowed from Visit 14 through Visit 58, as long as they are not two or more successive skips or skipping of the following Visits for examination (Visits19, 25, 31, 37, 43, 49, and 55).

Table 7-3 Acceptable Time Window

Study Period (Study Visit)	Acceptable Time Window	Study Procedures
Screening Period	Before the day of enrollment	• DNA typing for HLA-A<br>• Sample collection for antigen expression assessment<br>• Height
	Within 28 days before the day of enrollment	• Physical examination<br>• Weight<br>• ECG<br>• PS (ECOG)<br>• Pregnancy test<br>• Tumor evaluation (diagnostic imaging)
	Within 28 days before the day of enrollment and after 21days from the last administration of chemotherapy	• Routine laboratory test (hematology, blood chemistry, and urinalysis, specified in Table 7-2)
Treatment Period (Visit 1)	Prior to initial administration	• Enrollment in the study<br>• Physical examination<br>• Vital signs<br>• Sample collection for CTL analysis and repertoire analysis<br>• QOL questionnaires
Treatment Period (Visit 2 to 18)	± 3 days	• Administration<br>• Physical examination<br>• PS (ECOG)<br>• Vital signs
	± 7 days	• ECG<br>• Sample collection for CTL analysis and repertoire analysis<br>• Tumor evaluation (diagnostic imaging)

Study Period (Study Visit)	Acceptable Time Window	Study Procedures
		• Routine laboratory test (hematology, blood chemistry, and urinalysis, specified in Table 7-2)<br>• QOL questionnaires
Treatment Period (Visit 19 to 58)	± 3 days	• Administration<br>• Physical examination<br>• PS (ECOG)<br>• Vital signs
	± 7 days	• ECG<br>• Routine laboratory test (hematology, blood chemistry, and urinalysis, specified in Table 7-2)<br>• QOL questionnaires
	± 14 days	• Sample collection for CTL analysis and repertoire analysis<br>• Tumor evaluation (diagnostic imaging)
Treatment Period (Visit 59)	± 2 days	• Physical examination<br>• Weight<br>• PS (ECOG)<br>• Vital signs
	Within 2 days before Day 729 or 7 days after Day 729	• ECG<br>• Pregnancy test<br>• Sample collection for CTL analysis and repertoire analysis<br>• Tumor evaluation (diagnostic imaging)<br>• Routine laboratory test (hematology, blood chemistry, and urinalysis, specified in Table 7-2)<br>• QOL questionnaires
Treatment Period (Discontinuation)	Within 28 days after the day of Discontinuation	• Physical examination<br>• Weight<br>• PS (ECOG)<br>• Vital signs<br>• ECG<br>• Routine laboratory test (hematology, blood chemistry, and urinalysis, specified in Table 7-2)<br>• Pregnancy test<br>• Sample collection for CTL analysis and repertoire analysis<br>• Tumor evaluation (diagnostic imaging)<br>• Sample collection for antigen expression assessment<br>• QOL questionnaires

8. PLANNED STATISTICAL METHODS

8.1 General Considerations

The statistical analysis will be performed by the sponsor. The detailed statistical analysis methods will be specified in a statistical analysis plan according to this section of the study protocol.

Unless otherwise noted, continuous variables will be summarized by using the number of non-missing observations, arithmetic mean, standard deviation, median, minimum, and maximum values as descriptive statistics; categorical variables will be summarized by using the frequency count and the percentage of patients in each category as descriptive statistics.

Statistical test for the primary analysis will be performed at the 0.05 significance level using one-sided test.

All patient study data, including data not appearing in tables, will be presented in by-patient data listings. In general, all tables will be presented by treatment group. Individual patient data, and any derived data will be presented by treatment and patient. All analyses and tabulations will be performed by using the SAS Version 9.2 or higher.

8.2 Determination of Sample Size

- S-588410 Group

The CTL induction rate within 12 weeks after initial dose is the primary endpoint in this study. As an anti-malignant tumor drug, the compound should provide a CTL induction rate of at least 50% in the S-588410 Group. In this study, the null hypothesis is that the CTL induction rate is 50% or below and the alternative hypothesis is that this rate exceeds 50%. The CTL induction rates were 67% (4/6 patients who received 1 mg each of DEPDC1-derived peptide and MPHOSPH1-derived peptide) in the Translational Research (TR) study, 93% (14/15 patients in the 1 mg group) and 83% (15/18 patients in the 2 mg group) in the Ph1/2 study of S-288310 which contains S-288301 and S-288302. Based on these results, the combined CTL induction rate was estimated to be 88.3% (99%confidence interval [CI]: 75.3% to 100.0%) by meta-analysis. Assuming a CTL induction rate of 75% in the present study, the study requires 35 patients to have a 90% power at a one-sided 5% significance level. In anticipation of dropout of 15%, the total target sample size is 42 patients.

- Observation Group

As an exploratory analysis, progression free survival curves and overall survival curves will be constructed for the Observation Group as well as for the S-588410 Group. The maximum sample size of 42 patients is required for the Observation Group to enable the estimation with a similar accuracy to the S-588410 Group.

8.3 Analysis Populations

The modified intention-to-treat (mITT) population will be the primary population for the primary efficacy endpoint. The intention-to-treat (ITT) population will be the analysis

population for secondary efficacy endpoints. The per-protocol set (PPS) will be used for sensitivity analyses.

- **ITT population** includes all enrolled patients.
- **mITT population** includes all patients who are included in the ITT population and who have a CTL activity measurement at baseline and at least 1 CTL activity measurement after the initiation of study drug administration. Patients whose CTL activities are not available to all of 5 antigens at baseline or are already extremely strong (3+) to all of 5 antigens at baseline are excluded from the mITT population.
- **Per-protocol set (PPS)** includes all patients who are included in the mITT population and do not meet any of the following conditions:
  - Patients with any protocol inclusion or exclusion violations
  - Patients who have insufficient treatment compliance of study drug
  - Patients with violations of restrictions for concomitant drug and concomitant therapy.
- **Safety population** includes all enrolled patients in the S-588410 Group who receive at least 1 actual dose of S-588410 and all patients enrolled in the Observation Group.

8.4 Handling of Missing Data

Missing data will not be replaced. All analyses will be based on observed cases.

8.5 Patient Dispositions

Among the enrolled patients in each group, the number and percent of patients who complete the study and the number who prematurely discontinue the study will be summarized. In addition, reasons leading to study discontinuation will be summarized for each group. The number and percent of patients for the enrolled subjects included in each analysis population will also be presented.

8.6 Demographics and Baseline Characteristics

Demographic and baseline characteristics will be summarized with descriptive statistics by the treatment group for the safety population, the ITT population, and the mITT population.

8.7 Prior Therapies

Prior therapies for drugs will be coded using the World Health Organization (WHO) Drug Dictionary. Patients who received prior therapy(ies) will be listed for the safety population and the ITT population.

8.8 Concomitant Therapies

Concomitant therapies for drugs will be coded using the WHO Drug Dictionary. Patients who received concomitant therapy(ies) will be listed for the safety population and the ITT population.

8.9 Efficacy Analyses

8.9.1 Primary Efficacy Endpoint

The primary efficacy endpoint is the CTL induction rate within 12 weeks after initial dose, defined as the proportion of patients who show in vitro CTL induction to at least any of the 5 antigens (DEPDC1, MPHOSPH1, URLC10, CDCA1, and KOC1). The CTL induction is defined as the increase in the CTL activity at any point after baseline (eg, from [−] to [2+], from [+] to [2+]). Baseline is defined as the CTL measurement taken prior to dosing on the date of the first dose.

8.9.2 Secondary Efficacy Endpoints

The secondary efficacy endpoints include the following variables:

- CTL induction rate within 1 year after initial dose, defined as the proportion of patients who show in vitro CTL induction to at least any one of the 5 antigens.
- Response rate (RR), defined as the proportion of patients who are assessed as CR or PR by using RECIST version 1.1 and irRC, respectively.
- Disease control rate (DCR), defined as the proportion of patients who are assessed as CR, PR, or SD by using RECIST version 1.1 and irRC, respectively.
- Any response rate in image analysis such as tumor cavitation, defined as the proportion of patients who show any tumor change in image analysis (eg, tumor cavitation).
- PFS, defined as the time interval from the date of enrollment to the date of progression (in order of the following priority; PD by central review based on RECIST version 1.1, withdrawal due to aggravation of the target disease, or progression of the target disease by the investigator in Vital Status Follow-up Form) or death due to any cause, whichever occurs first.
- OS, defined as the time interval from the date of enrollment to the date of death due to any cause or the date of last follow-up.
- Change in QOL, defined as change from baseline in the global health status, the function scales, and the symptom scales on the EORTC QLQ-C30 questionnaire, and the index value and the EQ VAS on the EQ-5D-5L questionnaire, respectively. Baseline is defined as the value obtained at Visit 1 (pre-dose).

8.9.3 Analyses of Efficacy Endpoints

8.9.3.1 CTL Induction Rate for the S-588410 Group

For the mITT population, CTL induction rate and the associated 90% CI will be estimated by visit. As the primary analysis, one-sided binomial test where the null hypothesis is that the CTL induction rate within 12 weeks is equal to 0.5 or less will be performed at a significance level of 0.05. The 90% CI will then be calculated by using the Clopper-Pearson method.

The number of antigens with CTL induction will be tabulated by visit.

As a sensitivity analysis, the primary efficacy endpoint will be analyzed using the PPS.

8.9.3.2 Tumor Response

- Response rate and disease control rate using RECIST version 1.1 and irRC:
  For the ITT population, the RR and the DCR using RECIST version 1.1 and irRC will be determined, and the associated 90% CIs will be calculated for the S-588410 Group and the Observation Group, respectively.
- Any response rate in image analysis such as tumor cavitation:
  For the ITT population, the response rate in image analysis will be determined, and the associated 90% CI will be calculated for the S-588410 Group and the Observation Group, respectively.

8.9.3.3 Progression Free Survival

For the ITT population, the PFS will be estimated with the Kaplan-Meier method and the median PFS and the associated 90% CI will be presented for the S-588410 Group and the Observation Group, respectively.

8.9.3.4 Overall survival

For the ITT population, the OS will be estimated with the Kaplan-Meier method and the median OS and the associated 90% CI will be presented for the S-588410 Group and the Observation Group, respectively.

8.9.3.5 QOL

For the ITT population, the change from baseline in the global health status, the function scales, and the symptom scales on the EORTC QLQ-C30 questionnaire, and the index value and the EQ VAS on the EQ-5D-5L questionnaire will be summarized by visit for the S-588410 Group and the Observation Group, respectively.

8.10 Safety Analyses

8.10.1 Adverse Events

AEs will be classified by system organ class and preferred term using Medical Dictionary for Regulatory Activities (MedDRA) Version 16.0 or higher. Of reported AEs on the CRF, TEAEs will be used in the S-588410 Group and AEs will be used in the Observation Group for safety analyses. The definition of TEAE is described in Section 7.6.11.

For the S-588410 Group, the number and the percentage of patients who experienced TEAEs will be summarized. For the overall summary of TEAE, the number and the percentage of patients who have experienced death, serious TEAEs, Grade 3 or higher TEAEs, and TEAEs that lead to withdrawal of study drug will also be summarized. The number of those TEAEs, which are counted by cases reported, will also be presented. ADRs will be summarized by the same as TEAE category of overall summary. For the summary of TEAE by system organ class and preferred term, the number of patients who have experienced TEAEs will be presented for the S-588410 Group with the percent of patients. The summary by severity and the summary by outcome will be presented by system organ class and preferred term.

For the S-588410 Group, the number and the percentage of patients who experienced local reaction-related AEs as the injection sites will be summarized. In a similar fashion, eosinophilic pneumonia-related AEs, flulike symptoms-related AEs, and anaphylactic reaction-related AEs will be tabulated, respectively.

For the Observation Group, the number and the percentage of patients who experienced AEs will be summarized. For the overall summary of AE, the number of patients who have experienced death, SAEs will be presented with the percent of patients who have experienced their AEs. The number of those AEs, which are counted by cases reported, will also be presented. For the summary of AE by system organ class and preferred term, the number of patients who have experienced AEs will be presented for the Observation group with the percent of patients. The summary by severity will be presented by system organ class and preferred term.

All AEs including AEs that have presented prior to or after the first dose of the study drug will be listed.

8.10.2 Injection Site Reactions

For the S-588410 Group, the number of patients for each sign of injection site reactions will be summarized at each scheduled time point measured after enrollment.

Injection site reactions for signs will be listed.

8.10.3 Performance Status

For the S-588410 Group, PS (ECOG) data at baseline and at each scheduled time point measured after enrollment will be cross-tabulated. Baseline is defined as the last value obtained between Screening Visit and Visit 1 (pre-dose).

PS will be listed for the S-588410 Group and the Observation Group, respectively.

8.10.4 Vital Signs

For the S-588410 Group, summary statistics for vital signs will be presented for each scheduled time point measured after enrollment and for the change from baseline to each time point. Baseline is defined as the last value obtained between Screening Visit and Visit 1 (pre-dose).

Vital signs will be listed for the S-588410 Group and the Observation Group, respectively.

8.10.5 Electrocardiogram

For the S-588410 Group, ECG findings at baseline and at each scheduled time point measured after enrollment will be cross-tabulated. Baseline is defined as the last value obtained between Screening Visit and Visit 1 (pre-dose).

ECG findings will be listed for the S-588410 Group and the Observation Group, respectively.

8.10.6 Clinical Laboratory Analysis

For the S-588410 Group, summary statistics for laboratory test data will be presented for each scheduled time point measured after enrollment and for the change from baseline to each time point. Qualitative laboratory test data at baseline and at each scheduled time point measured after enrollment will be cross-tabulated. Baseline is defined as the last value obtained between Screening Visit and Visit 1 (pre-dose).

Clinical laboratory test data will be listed for the S-588410 Group and the Observation Group, respectively.

8.11 Interim Analysis

No interim efficacy analysis for a judgment of study discontinuation or a change of study design is planned for this study. Statistical analysis will be performed after all enrolled patients complete Treatment Period/Observation Period.

9. ADMINISTRATIVE CONSIDERATIONS

9.1 Investigators and Study Administrative Structure

Sponsor:	Shionogi & Co., Ltd.<br>(Head Office) 3-1-8 Doshomachi, Chuo-ku, Osaka 541-0045, Japan
Sponsor's representative:	[redacted], General manager,<br>Clinical Research Department, Shionogi & Co., Ltd.
Sponsor's study manager:	[redacted]<br>Clinical Research Department, Shionogi & Co., Ltd.<br>12th floor, Hankyu Terminal Bldg., 1-4, Shibata 1 -chome, Kita-ku, Osaka 530-0012, Japan<br>TEL: [redacted]<br>[redacted]<br>Senior Manager, Shionogi Ltd.<br>5th floor, 33 Kingsway, Holborn, London WC2B 6UF, United Kingdom<br>TEL: [redacted]
Sponsor's chief medical officer:	[redacted]<br>Medical Science Department, Shionogi & Co., Ltd.
Medical officer:	[redacted], Japan<br>TEL: [redacted]<br><br>[redacted] Japan<br>TEL: [redacted]
Medical monitor:	[redacted]<br>Medical Consultant Oncology, Shionogi Ltd.<br>5th floor, 33 Kingsway, Holborn, London WC2B 6UF, United Kingdom<br>TEL: [redacted]<br>FAX: [redacted]
Investigator and study center:	See a separate list
Study monitoring:	See a separate list
EU legal representative:	[redacted]<br>Head of Development, Europe,<br>Shionogi Ltd.<br>5th floor, 33 Kingsway, Holborn, London WC2B 6UF, United Kingdom<br>TEL: [redacted]<br>FAX: [redacted]

Contract Research Organization (monitoring for Japanese study sites):	[redacted], Japan<br>TEL: [redacted]
Central laboratory and company for delivery of samples (for Japanese study sites):	[redacted] Japan<br>TEL: [redacted]<br>FAX: [redacted]
Central laboratory and company for delivery of samples (for EU study sites):	[redacted] United Kingdom<br>TEL: [redacted]<br>FAX: [redacted]
Laboratory for antigen expression testing of tumor tissue, laboratory for preparation of cDNA, and company for storage of samples for TCR repertoire analysis:	[redacted] Japan<br>TEL: [redacted]<br>FAX: [redacted]
Laboratory for specific CTL measurement:	[redacted]<br>Medical Research Laboratories, Shionogi & Co., Ltd.<br>1-1, Futaba-cho 3-chome, Toyonaka, Osaka, 561-0825, Japan<br>TEL: [redacted]<br>FAX: [redacted]
Laboratory for TCR repertoire analysis:	[redacted]<br>United States<br>TEL: [redacted]
Central Imaging:	[redacted],<br>United States<br>TEL: [redacted]<br>FAX: [redacted]

9.2 Institutional Review Board or Institutional Ethics Committee Approval

The IRB/IEC will safeguard the rights, safety, and well-being of the patients by reviewing the following study documents: the study protocol, informed consent form, written information on patient recruitment procedures (if applicable), other written information given to the patients, IB, safety updates, annual progress reports (if applicable), and any significant revisions to these documents. The investigator or the sponsor will provide these study documents to the IRB/IEC. The IRB/IEC will be appropriately constituted in accordance with International Conference on Harmonisation (ICH) Good Clinical Practice (GCP), and local requirements, as applicable. The study will be undertaken only after IRB/IEC has given full approval and the sponsor has received a copy of the approval. The approval letter must be dated and must clearly identify the documents being approved.

Amendments to the study protocol will be subject to the same requirements as the initial review. The investigator will submit all periodic reports and updates as required by the IRB/IEC. The investigator will inform the IRB/IEC of any reportable AEs.

9.3 Ethical Conduct of the Study

The study will be conducted in accordance with all appropriate regulatory requirements and under protocol approved by an IRB/IEC. The study will be conducted in accordance with current ICH GCP, all appropriate patient privacy requirements, and the ethical principles that are outlined in the Declaration of Helsinki.

9.4 Patient Information and Consent

The investigator will generate an informed consent form for the study. The consent form will include all the elements required by the ICH GCP and any additional elements required by local regulations and will be reviewed and approved by the appropriate IRB/IEC before use. The investigator or subinvestigator will explain the nature, purpose and methods, reasonable anticipated benefits and potential hazards of the study to the patient in simple terms by using the consent form before the patient is entered the study. The method of obtaining and documenting informed consent will comply with ICH GCP and all applicable regulatory requirement(s).

The sponsor will provide the investigators with a proposed informed consent form that complies with the ICH GCP guidelines and regulatory requirements. The sponsor must agree to any changes to the proposed consent form suggested by the investigator prior to submission to the IRB/IEC, and the IRB/IEC approved version must be provided to the site monitor after IRB/IEC approval.

9.5 Patient Confidentiality

Procedures for protecting patient privacy must adhere to applicable data privacy laws and regulations. In order to maintain patient privacy, all CRFs, study drug accountability records, study reports, and communications will identify the patient by the patient number. The investigator will grant site monitor(s) and auditor(s) of the sponsor or

designee and regulatory authority(ies) access to all source documents for verification of data collected on the CRFs and for verification of the data collection process. The patient's confidentiality will be maintained and will not be made publicly available to the extent permitted by the applicable laws and regulations. The investigator and the sponsor are responsible for ensuring that sensitive information is handled in accordance with local requirements (eg, HIPAA). Appropriate consent and authorizations for use and disclosure and/or transfer (if applicable) of protected information must be obtained.

Data on patients collected on CRFs during the study will be documented in an anonymous fashion and the patient will only be identified by the patient number. In the emergent or rare event that for safety or regulatory reasons it is necessary to identify a patient, the sponsor, and the investigator are bound to keep this information confidential.

9.6 Study Monitoring

The sponsor or designee will monitor the study to ensure that the study is conducted in accordance with GCP requirements and the study protocol. The study monitoring will be performed by a representative of the sponsor (site monitor) through on-site monitoring visits as frequently as necessary and frequent communications (e-mail, letter, telephone, and fax). The site monitor will review data recorded on the CRFs, verify the CRFs entries with direct access to source documents, collect any safety/efficacy information on patients, verify that amounts of unused study drug are accurate, and check retention of source documents and essential documents.

9.7 Case Report Forms and Source Documents

9.7.1 Case Report Forms

The sponsor or designee will supply CRFs. CRFs for each patient signing informed consent will be provided within 72 hours after each patient signs informed consent. Historical information and study data, which are specified by the study protocol, will be recorded on CRFs by the investigator. All patient data from study visits must be collected on source documents and are promptly entered in the CRFs in accordance with the specific instructions given. CRF entries will be performed by an investigator, subinvestigator, and study coordinator who are authorized in document.

When queries will be generated to the participating medical institutions for resolution by the sponsor or designee, CRF data will be changed or a response will be recorded in accordance with the specific instructions given. The investigator must ensure that data reported on the CRF is accurate, complete, legible, and timely and sign the CRFs to verify the integrity of the data recorded.

A list of the reference ranges for all laboratory tests to be undertaken will be a part of the documentation to be collected prior to the initiation of study. The list of reference ranges for all laboratory tests should be updated during the study. If a central laboratory has been selected to conduct any or all tests, it is essential for all samples to be analyzed at that laboratory.

9.7.2 Source Documents

Source documentation supporting the CRF data should indicate the patient's participation in the study and should document the dates and details of study procedures, AEs, and patient status. However, the following data will be allowed as data which can be directly recorded on a CRF.

- reason for use of prior therapy or concomitant therapy
- severity, seriousness, causal relationship to the study drug of AE
- any comments inserted into CRF

The following data will be automatically calculated in the CRFs.

- Age

The investigator must maintain source documents such as laboratory reports, and complete medical history and physical examination reports. All the source documents are accessible for verification by the site monitor, auditor, IRB/IEC, inspections of regulatory authority. Direct access to these documents must be guaranteed by the investigator, subinvestigator, or study coordinator, who must provide support at all times for these activities. For all sources of original data required to complete the CRF, the nature and location of the source documents will be identified by the sponsor and the site staff. If electronic records are maintained at the medical institution, the method of verification must be specified in document within the medical institution.

9.7.3 External Data

The following data will be reported in separate documents from CRFs.

- Results of antigen expression testing
- Results of routine laboratory tests (see also Table 7-2)
- Results of specific CTL analysis
- Results of tumor evaluation by central review
- Survey results from Vital Status Follow-up Form
- Results of TCR repertoire analysis

9.8 Committees

9.8.1 Case Review Committee

The sponsor will review all data reported on CRFs of all enrolled patients with the medical officer before database lock. The sponsor will decide patients that will be included in the analysis population based on the definition of analysis populations specified in the study protocol and the results at the case review committee. The case review committee also evaluates whether medical decisions of the investigators were appropriate for important data affecting the safety and efficacy endpoints.

9.8.2 Independent Data Monitoring Committee

The independent data monitoring committee (IDMC) will assess the safety and efficacy

data and recommend whether or not to continue the study in accordance with manual.

The IDMC meeting will be held once the safety data from the first 6 patients who received 4 weeks of S-588410.

9.9 Termination or Suspension of the Study

9.9.1 Termination or Suspension of the Entire Study

The sponsor may prematurely terminate or suspend the study at any time for the following reasons:

- Ensuring safety of the study is difficult due to safety concerns (eg, occurrence of many serious ADRs).
- Achieving the purpose of the study is considered impossible (eg, interim data suggesting lack of efficacy/safety, inadequate recruitment of patients).

If the study is prematurely terminated or suspended, the sponsor should promptly inform the investigators. The investigator or subinvestigator should promptly inform the participating patients and change the study treatment to other appropriate therapy(ies).

For withdrawal criteria for individual patients, see Section 7.7.

9.9.2 Termination or Suspension of the Study by Medical Institution

The investigator may prematurely terminate or suspend only the study in the medical institution with agreement of the sponsor at any time when the investigator considers that ensuring safety of the study is difficult due to safety concerns (eg, occurrence of many SAEs).

The sponsor may request the investigator to prematurely terminate or suspend the study in the medical institution at any time when major violations/deviations of protocol, other procedures, and GCP guidelines were not improved by the investigator.

If the study is prematurely terminated or suspended, the investigator or subinvestigator should promptly inform the corresponding IRB/IEC and participating patients and change the study treatment to other appropriate therapy(ies).

9.10 Protocol Deviations and Modifications

The investigator will conduct the study in compliance with the study protocol provided by the sponsor and approval/favorable opinion given by the IRB and the regulatory authority(ies). Modifications to the study protocol should not be performed without agreement of both the investigator and the sponsor. Changes to the study protocol will require written IRB approval/favorable opinion prior to implementation, except when the modification is needed to eliminate an immediate hazard(s) to patients.

The investigator or subinvestigator should document any deviation from the study protocol and the reason. If the investigator performs a deviation from the study protocol or a change of the study protocol to eliminate an immediate hazard(s) to patients, the

record should be immediately submitted to the sponsor, the medical institution, and the IRB by the investigator and the IRB will provide expedited review and approval/favorable opinion. After the investigator obtained approval/favorable opinion of the IRB, the investigator should obtain written permission of the medical institution and written agreement of the sponsor.

When deviation from the study protocol is required to eliminate immediate hazard(s) to patients, the investigator will contact the sponsor, if circumstances permit, to discuss the planned course of action. Any deviations from the study protocol must be fully documented on source documentation.

9.11 Data Management

The sponsor will be responsible for data management and data analysis. These procedures are specified in a separate document.

9.12 Retention of Data

The study documents must be maintained as specified in the ICH GCP and as required by the applicable regulatory requirements. The investigator and study center should take measures to prevent accidental or premature destruction of these documents.

If the sponsor is granted manufacturing and marketing approval for the drug, the sponsor will promptly notify the head of the study center in writing.

Records will be retained for any of the following periods:

- until the approval day of manufacturing/marketing on the study drug or 3 years after the decision day on the discontinuation of development.
- 3 years after the decision day on the discontinuation or completion of the study, whichever is later.

However, the duration of retention may be prolonged with agreement with the sponsor. If the investigator withdraws from the responsibility of keeping the study records, custody must be transferred to a person willing to accept the responsibility.

9.13 Quality Control and Assurance

The sponsor or designee will implement and maintain quality control and quality assurance procedures with written standard operating procedures to ensure that the study is conducted and data are generated, documented, and reported in compliance with the study protocol, ICH GCP, and applicable regulatory requirements.

This study will be conducted in accordance with the provisions of the Declaration of Helsinki and all revisions thereof; in accordance with the ICH GCP and as required by the applicable regulatory requirements.

Necessary training for the study will be provided to investigator's meeting and study center personnel prior to the initiation of the study.

9.14 Publication and Disclosure Policy

All information regarding S-588410 supplied by the sponsor to the investigator is privileged and confidential. The investigator agrees to use this information to accomplish the study and will not use it for other purposes without consent from the sponsor. It is understood that there is an obligation to provide the sponsor with complete data obtained during the study. The information obtained from the clinical trial will be used toward the development of S-588410 and may be disclosed to regulatory authority(ies), other investigators, corporate partners, or consultants as required.

The sponsor will retain ownership of all data. All proposed publications based on the study will be subject to the sponsor's approval requirements.

The key design elements of this study protocol will be posted in a publicly accessible database.

10. REFERENCE LIST

1. Jemal A, Bray F, Center MM, Ferlay J, Ward E, Forman D. Global cancer statistics. CA Cancer J Clin 2011; 61: 69-90.
2. The Japanese Urological Association, ed. Clinical Practice Guidelines in Bladder Cancer [in Japanese]. Igaku Tosho Shuppan & Co., Ltd.; March 2009 edition.
3. National Cancer Institute Physician Data Query (NCI-PDQ). Bladder Cancer Treatment. Date of last modified, 12 July 2012.
4. European Association of Urology. Guidelines on Bladder Cancer Muscle-invasive and Metastatic. Update February 2012.
5. The Committee for Establishment of the Clinical Practice Guidelines for the Management of Bladder Cancer and the Japanese Urological Association. Evidence-based clinical practice guidelines for bladder cancer (Summary – JUA 2009 Edition). International Journal of Urology 2010: 17, 102–24.
6. Sonpavde G, Sternberg CN, Rosenberg JE, et al. Second-line systemic therapy and emerging drugs for metastatic transitional-cell carcinoma of the urothelium. Lancet Oncol 2010;11(9):861-70.
7. CHMP Assessment Report for Javlor. European Public Assessment Report. European Medicines Agency. 10 August 2009.
8. T Fujioka. Tumor-specific vaccine therapy using epitope peptide derived from a tumor antigen gene for the upper urinary tract cancer. Reports under Grants-in-Aid for Scientific Research 2 May 2011.
9. Longo DL. Tumour heterogeneity and personalized medicine. N Engl J Med 2012; 366(10): 956-7.
10. Cecco S, Muraro E, Giacomin E, et al. Cancer vaccines in phase II/III clinical trials: state of the art and future perspectives. Curr Cancer Drug Targets 2011; 11(1): 85-102.
11. Petros D. Grivas, Stephanie Daignault, Scott T. Tagawa, et al. Double-Blind, Randomized, Phase 2 Trial of Maintenance Sunitinib Versus Placebo After Response to Chemotherapy in Patients With Advanced Urothelial Carcinoma. Cancer 2013; Article first published online 18 NOV 2013.
12. Study Title: A Phase II/III, Randomised, Two-Arm, Comparison of Maintenance Lapatinib Versus Placebo After First-Line Chemotherapy in Patients With HER1 and/or HER2 Overexpressing Locally Advanced or Metastatic Bladder Cancer. http://clinicaltrials.gov/show/NCT00949455
13. Study Title: Randomized Phase II Study of Vinflunine as Maintenance Monotherapy in Patients With Advanced or Metastatic Urothelial Cancer That Obtains Clinical Benefit of the First Line With Cisplatin-gemcitabine Combination. http://clinicaltrials.gov/show/NCT01529411
14. Guidance for Industry: Clinical Considerations for Therapeutic Cancer Vaccines. October 2011.
15. Oken MM, Creech RH, Tormey DC, et al. Toxicity and response criteria of the Eastern Cooperative Oncology Group. Am J Clin Oncol 1982;5(6):649-55.
16. Rothenberg ME. Eosinophilia. N Engl J Med 1998; 338:1592-1600.
17. Guidance for Industry: Clinical Trial Endpoints for the Approval of Cancer Drugs and Biologics. May 2007.
18. Guideline for Clinical Evaluation of Anti-Malignant Tumour Agents [in Japanese]. Ministry of Health, Labour and Welfare. November 1, 2005.

19. European Medicines Agency. Guideline on the evaluation of anticancer medicinal products in man, published in January 2013.

Appendix 1 Time and Events Schedule in the S-588410 Group

	Screening	Before Dosing	Treatment Period 104 weeks																				Discon-tinuation	Follow-up Assess-ment
Study Visit	-	1		2	3	4	5	6	7	8	9	10	11	12	13	14	···	31	···	55	···	59	-	-
Study Week	-	0		1	2	3	4	5	6	7	8	9	10	11	12	14	···	48	···	96	···	104	-	-
Study Day	−28 to −1	1		8	15	22	29	36	43	50	57	64	71	78	85	99	···	337	···	673	···	729	-	-
Informed consent[a]	X																···		···		···			
Inclusion/exclusion criteria	X																···		···		···			
Baseline patientcharacteristics	X																···		···		···			
HLA-A genotyping[b]	X																···		···		···			
Antigen Expression Testing	X																···		···		···		X[c]	
Height	X																···		···		···			
Weight	X																···		···		···	X	X[c]	
PS (ECOG)	X	X		X					X						X		Every 12 weeks from Visit 13			X	···	X	X[c]	
Vital signs		X		X	X	X	X	X	X	X	X	X	X	X	X	X	The same Visit as for administration from Visit 14					X	X[c]	
ECG	X														X		Every 12 weeks from Visit 13			X	···	X	X[c]	
Routine laboratory tests	X[d]			X					X						X		Every 12 weeks from Visit 13			X	···	X	X[c]	
HIV, HBV, HCV tests	X																···		···		···			
Pregnancy test[e]	X																···		···		···	X	X[c]	
Administration			X	X	X	X	X	X	X	X	X	X	X	X	X	X	Every 2weeks from Visit 14							
Assessment of Injection Site				X	X	X	X	X	X	X	X	X	X	X	X	X	···		···		···		X[c]	
Physical examination	X	X	X[f]	X	X	X	X	X	X	X	X	X	X	X	X	X	The same Visit as for administration from Visit 14					X	X[c]	

	Screening	Before Dosing	Treatment Period 104 weeks																			Discon-tinuation	Follow-up Assess-ment
Study Visit	-	1	2	3	4	5	6	7	8	9	10	11	12	13	14	···	31	···	55	···	59	-	-
Study Week	-	0	1	2	3	4	5	6	7	8	9	10	11	12	14	···	48	···	96	···	104	-	-
Specific CTL measurement		X								X				X		Every 12 weeks from Visit 13	X	···		···	X	X[c]	
Repertoire analysis		X								X				X		Every 12 weeks from Visit 13	X	···		···	X	X[c]	
Tumor evaluation	X													X		Every 12 weeks from Visit 13			X	···	X	X[c]	
QOL questionnaires		X				X								X		Every 12 weeks from Visit 13			X	···	X	X[c]	
Evaluation of survival[g]			← X →																			X	← X →
Reporting Post- therapy for target disease[h]																							← X →
Adverse event	← X →																					X	

HBV, hepatitis B virus; HCV, hepatitis C virus; HIV, human immunodeficiency virus; HPV, human papilloma virus

a. Informed consent must be obtained before screening.
b. HLA-A genotyping will be performed after informed consent has been obtained, in patients for whom the HLA-A type has not yet been identified.
c. Perform the test if possible.
d. Within 28 days before the day of enrollment and after 21days from the last administration of chemotherapy.
e. Only for female patients with the exception of patients who are postmenopausal (at least 2 years since their last regular menstrual period without any other medical reason) or who are surgically sterile by hysterectomy and/or bilateral oophorectomy, or by tubal ligation
f. Patients will rest for at least 30 minutes after the initial dose, and will be closely monitored under a physician's supervision.
g. Overall survival and progression-free survival will be investigated every 6 months for up to 3 years after the date the last patient is enrolled.
h. Subsequent therapy for target disease will be investigated every 6 months for up to 3 years after the date the last patient is enrolled.

Appendix 2 Time and Events Schedule in the Observation Group

	Screening	Observation Period 104 weeks																Discon-tinuation	Follow-up Assess-ment
Study Visit	-	1	2	3	4	5	6	7	8	9	10	11	12	13	···	55	59	-	-
Study Week	-	0	1	2	3	4	5	6	7	8	9	10	11	12	···	96	104	-	-
Study Day	−28 to −1	1	8	15	22	29	36	43	50	57	64	71	78	85	···	673	729	-	-
Informed consent[a]	X														···				
Inclusion/exclusion criteria	X														···				
Baseline patient characteristics	X														···				
HLA-A genotyping[b]	X														···				
Antigen Expression Testing	X														···			X[c]	
Height	X														···				
Weight	X														···		X	X[c]	
PS (ECOG)	X	X						X						X	Every 12 weeks from Visit 13	X	X	X[c]	
Vital signs		X				X		X						X	Every 12weeks from Visit 13	X	X	X[c]	
ECG	X													X	Every 12 weeks from Visit 13	X	X	X[c]	
Routine laboratory tests	X[d]							X						X	Every 12 weeks from Visit 13	X	X	X[c]	
HIV, HBV, HCV tests	X														···				
Pregnancy test[e]	X														···				
Physical examination	X	X				X		X						X	Every 12 weeks from Visit 13	X	X	X[c]	
Tumor evaluation	X													X	Every 12 weeks from Visit 13	X	X	X[c]	
QOL questionnaires		X				X								X	Every 12 weeks from Visit 13	X	X	X[c]	
Evaluation of survival[f]		← X →																X[c]	← X →
Reporting Subsequent Therapy for Target disease[g]																			← X →
Adverse event	← X →																	X	

HBV, hepatitis B virus; HCV, hepatitis C virus; HIV, human immunodeficiency virus; HPV, human papilloma virus

The patients in the Observation Group do not need to visit the study sites at Study Visits in shaded areas.

a. Informed consent must be obtained before screening.

b. HLA-A genotyping will be performed after informed consent has been obtained, in patients for whom the HLA-A type has not yet been identified.

c. Perform the test if possible.

d. Within 28 days before the day of enrollment and after 21days from the last administration of chemotherapy.

e. Only for female patients with the exception of patients who are postmenopausal (at least 2 year since their last regular menstrual period without any other medical reason) or who are surgically sterile by hysterectomy and/or bilateral oophorectomy, or by tubal ligation.

f. Overall survival and progression-free survival will be investigated every 6 months for up to 3 years after the date the last patient is enrolled.

g. Subsequent therapy for target disease will be investigated every 6 months for up to 3 years after the date the last patient is enrolled.

Appendix 3 Management and Discontinuation Criteria for Abnormal Liver Function Tests

Management and Discontinuation Criteria for Abnormal Liver Function tests have been designed to ensure patient safety and evaluate liver event etiology. (See Guidance for Industry Drug-Induced Liver Injury: Premarketing Clinical Evaluation, FDA: 2009)

Abnormal Liver Chemistry Criteria:

The investigator or subinvestigator must review study patient laboratories to identify if any levels meet the following criteria:

a. AST or ALT >5 × ULN

b. AST or ALT >3 × ULN and total bilirubin >2 × ULN or PT-INR >1.5, if PT-INR measured.

c. AST or ALT >3 × ULN with signs or symptoms compatible with hepatitis or hypersensitivity (eg, fatigue, nausea, vomiting, right upper quadrant pain or tenderness, jaundice, fever, rash, eosinophilia [>5%])

Action to be taken by Investigator:

If any of abnormal liver chemistry criteria is met, the investigator or subinvestigator must do the following:

- Patients must be instructed to hold study medication immediately. The investigator or subinvestigator should not re-challenge the patient with the investigational product without consulting the sponsor.
- Following the initial observed elevation, every effort should be made to have the patient return to the clinic within 48 to 72 hours to repeat liver function chemistries and for further hepatic evaluation.
- Patients must be monitored 2 to 3 times per week until liver function chemistries (ALT, AST, ALP, total bilirubin) resolve, stabilize or return to within the normal range or to baseline levels.
- This event must be reported to the sponsor within 72 hours of learning after its occurrence on the Liver Event Form (refer to 7.6.8.2, for reporting destination).
- Consultation with a specialist such as a hepatologist is considered.
- Liver imaging (ie, ultrasound, MRI, CT) is considered.
- For criteria b, termed “Hy’s law“, the case must be reported as an SAE.

Follow-up Examination:

If any of abnormal liver chemistry criteria are met, the following assessments should be performed at the follow-up visit(s) and documented in the Liver Event Form:

- Clinical symptoms course

- Concomitant medications: OTC/herbal/dietary supplements (start and stop dates)
- Alcohol use
- Risk factors for nonalcoholic steatohepatitis (NASH) such as diabetes, obesity and hypertriglyceridemia
- Autoimmune hepatitis/cholangitis
- Wilson's disease
- Laboratory Assessments
  - Viral hepatitis serology
    - Hepatitis A IgM antibody
    - Hepatitis B surface antigen and Hepatitis B core antibody (IgM)
    - Hepatitis C RNA
    - Hepatitis E IgM antibody
    - Cytomegalovirus IgM antibody
    - Epstein-Barr viral capsid antigen IgM antibody
  - For patients with total bilirubin of >1.5 ULN, conjugated bilirubin should be measured
  - Complete blood count with differential to assess for eosinophilia

Re-starting Study Medication Criteria

For patients that meet the abnormal liver chemistry criteria, but only if they do not meet the "Drug Discontinuation Criteria Due to Abnormal Liver Chemistry Tests" may re-start the administration of study drug.

For patients with ALT >5 × ULN to ≤8 × ULN with elevations for less than two weeks, the study drug may be re-started at the discretion of the investigator and sponsor if both of the following conditions are met:

- Subsequent liver function chemistries are lower or unchanged
- No signs or symptoms consistent with hepatitis or hypersensitivity (eg, fatigue, nausea, vomiting, right upper quadrant pain or tenderness, jaundice, fever, rash or eosinophilia [>5%])

All patients that have met abnormal liver chemistry criteria must still be monitored 2 to 3 times per week until liver function chemistries (ALT, AST, alkaline phosphatase, and total bilirubin) resolve, stabilize or return to within the normal range or to baseline levels.

Drug Discontinuation Criteria Due to Abnormal Liver Chemistry Tests

Patients must be discontinued from the study as described below except that the investigator judges abnormal liver chemistry test values are due to target disease and discontinuation is not necessary:

- AST or ALT >8 × ULN, confirmed by follow-up testing (ie, initial abnormality is confirmed on subsequent testing), regardless of medical history or physical examination findings
- AST or ALT >5 × ULN, with elevations for more than 2 weeks or development of

concomitant signs or symptoms consistent with hepatitis or hypersensitivity (eg, fatigue, nausea, vomiting, right upper quadrant pain or tenderness, jaundice, fever, rash or eosinophilia [>5%])

- AST or ALT >3 × ULN and total bilirubin >2 × ULN or PT-INR >1.5, if PT-INR measured, confirmed by follow-up testing (ie, initial abnormality is confirmed on subsequent testing), regardless of medical history or physical examination findings.
- AST or ALT >3 × ULN with signs or symptoms compatible with hepatitis or hypersensitivity (eg, fatigue, nausea, vomiting, right upper quadrant pain or tenderness, jaundice, fever, rash or eosinophilia [>5%]), confirmed by follow-up testing (ie, initial laboratory abnormality is confirmed upon subsequent testing).
- AST or ALT >5 × ULN and the patient cannot be followed up weekly

Appendix 4 EORTC QLQ-C30 Questionnaires

ENGLISH

EORTC QLQ-C30 (version 3)

We are interested in some things about you and your health. Please answer all of the questions yourself by circling the number that best applies to you. There are no "right" or "wrong" answers. The information that you provide will remain strictly confidential.

Please fill in your initials: ↦↦↦↦

Your birthdate (Day, Month, Year): ↕□↕□↕↕↕□

Today's date (Day, Month, Year): 31 ↕□↕□↕↕↕□

	Not at All	A Little	Quite a Bit	Very Much
1. Do you have any trouble doing strenuous activities, like carrying a heavy shopping bag or a suitcase?	1	2	3	4
2. Do you have any trouble taking a long walk?	1	2	3	4
3. Do you have any trouble taking a short walk outside of the house?	1	2	3	4
4. Do you need to stay in bed or a chair during the day?	1	2	3	4
5. Do you need help with eating, dressing, washing yourself or using the toilet?	1	2	3	4

**During the past week:**	Not at All	A Little	Quite a Bit	Very Much
6. Were you limited in doing either your work or other daily activities?	1	2	3	4
7. Were you limited in pursuing your hobbies or other leisure time activities?	1	2	3	4
8. Were you short of breath?	1	2	3	4
9. Have you had pain?	1	2	3	4
10. Did you need to rest?	1	2	3	4
11. Have you had trouble sleeping?	1	2	3	4
12. Have you felt weak?	1	2	3	4
13. Have you lacked appetite?	1	2	3	4
14. Have you felt nauseated?	1	2	3	4
15. Have you vomited?	1	2	3	4
16. Have you been constipated?	1	2	3	4

Please go on to the next page

ENGLISH

**During the past week:**	**Not at All**	**A Little**	**Quite a Bit**	**Very Much**
17. Have you had diarrhea?	1	2	3	4
18. Were you tired?	1	2	3	4
19. Did pain interfere with your daily activities?	1	2	3	4
20. Have you had difficulty in concentrating on things, like reading a newspaper or watching television?	1	2	3	4
21. Did you feel tense?	1	2	3	4
22. Did you worry?	1	2	3	4
23. Did you feel irritable?	1	2	3	4
24. Did you feel depressed?	1	2	3	4
25. Have you had difficulty remembering things?	1	2	3	4
26. Has your physical condition or medical treatment interfered with your family life?	1	2	3	4
27. Has your physical condition or medical treatment interfered with your social activities?	1	2	3	4
28. Has your physical condition or medical treatment caused you financial difficulties?	1	2	3	4

For the following questions please circle the number between 1 and 7 that best applies to you

29. How would you rate your overall health during the past week?

1 2 3 4 5 6 7

Very poor Excellent

30. How would you rate your overall quality of life during the past week?

1 2 3 4 5 6 7

Very poor Excellent

© Copyright 1995 EORTC Quality of Life Group. All rights reserved. Version 3.0

Appendix 5 EQ-5D-5L Questionnaires

Under each heading, please tick the ONE box that best describes your health TODAY

MOBILITY

I have no problems in walking about ☐
I have slight problems in walking about ☐
I have moderate problems in walking about ☐
I have severe problems in walking about ☐
I am unable to walk about ☐

SELF-CARE

I have no problems washing or dressing myself ☐
I have slight problems washing or dressing myself ☐
I have moderate problems washing or dressing myself ☐
I have severe problems washing or dressing myself ☐
I am unable to wash or dress myself ☐

USUAL ACTIVITIES *(e.g. work, study, housework, family or leisure activities)*

I have no problems doing my usual activities ☐
I have slight problems doing my usual activities ☐
I have moderate problems doing my usual activities ☐
I have severe problems doing my usual activities ☐
I am unable to do my usual activities ☐

PAIN / DISCOMFORT

I have no pain or discomfort ☐
I have slight pain or discomfort ☐
I have moderate pain or discomfort ☐
I have severe pain or discomfort ☐
I have extreme pain or discomfort ☐

ANXIETY / DEPRESSION

I am not anxious or depressed ☐
I am slightly anxious or depressed ☐
I am moderately anxious or depressed ☐
I am severely anxious or depressed ☐
I am extremely anxious or depressed ☐

2

UK (English) v.2 © 2009 EuroQol Group. EQ-5D™ is a trade mark of the EuroQol Group

SUBMISSION

- We would like to know how good or bad your health is TODAY.
- This scale is numbered from 0 to 100.
- 100 means the best health you can imagine.
  0 means the worst health you can imagine.
- Mark an X on the scale to indicate how your health is TODAY.
- Now, please write the number you marked on the scale in the box below.

YOUR HEALTH TODAY = ☐

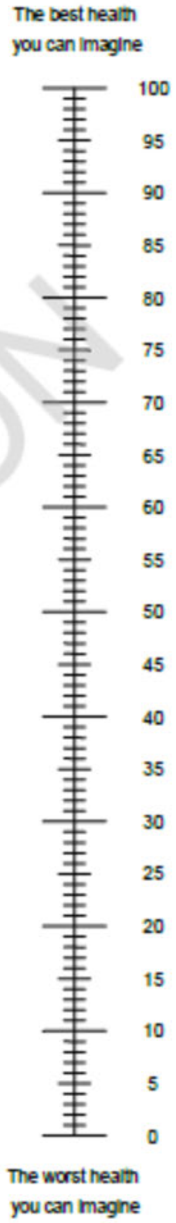

SUBMISSION

UK (English) v.2 © 2009 EuroQol Group. EQ-5D™ is a trade mark of the EuroQol Group

Appendix 6 Vital Status Follow-up Form

Vital Status Follow-up Form

A multicenter, open-label, phase 2 study of S-588410 as maintenance monotherapy after first-line platinum-containing chemotherapy in patients with advanced and/or metastatic bladder cancer

Institution	
Subject ID	
Disease has progressed?	□ Yes → Date of progression:_____/_____/_____(dd/MMM/yyyy)<br>Site of progression: □ Lymph node, □ Lung, □ Liver,<br>□ Other ( )<br>□ No → Date you last verified that the disease had not progressed:<br>_____/_____/_____(dd/MMM/yyyy)

Patient's vital status	□ Alive → Date you last verified that the patient was alive:<br>_____/_____/_____(dd/MMM/yyyy)<br>□ Deceased → Date of death:<br>_____/_____/_____(dd/MMM/yyyy)<br>□ Unknown → Date you last verified that the patient was alive:<br>_____/_____/_____(dd/MMM/yyyy)

If the patient is deceased:

Cause of death	□ Target disease<br><br>□ Other<br><br>If other, specify:
Autopsy findings	

Therapy other than surgical therapy for the target disease

#	Therapy name	Description*	Start date (dd/MMM/yyyy)	Stop date (dd/MMM/yyyy)	Best overall response	Response at the end of therapy	Reason for stopping therapy
1	□ GC therapy<br>□ MVAC therapy<br>□ Other<br>( )				□ CR<br>□ PR<br>□ SD<br>□ PD<br>□ Unknown	□ CR<br>□ PR<br>□ SD<br>□ PD<br>□ Unknown	□ Completion<br>□ Intolerance<br>□ Other<br>( )<br>□ Unknown
2	□ GC therapy<br>□ MVAC therapy<br>□ Other<br>( )				□ CR<br>□ PR<br>□ SD<br>□ PD<br>□ Unknown	□ CR<br>□ PR<br>□ SD<br>□ PD<br>□ Unknown	□ Completion<br>□ Intolerance<br>□ Other<br>( )<br>□ Unknown
3	□ GC therapy<br>□ MVAC therapy<br>□ Other<br>( )				□ CR<br>□ PR<br>□ SD<br>□ PD<br>□ Unknown	□ CR<br>□ PR<br>□ SD<br>□ PD<br>□ Unknown	□ Completion<br>□ Intolerance<br>□ Other<br>( )<br>□ Unknown
4	□ GC therapy<br>□ MVAC therapy<br>□ Other<br>( )				□ CR<br>□ PR<br>□ SD<br>□ PD<br>□ Unknown	□ CR<br>□ PR<br>□ SD<br>□ PD<br>□ Unknown	□ Completion<br>□ Intolerance<br>□ Other<br>( )<br>□ Unknown
5	□ GC therapy<br>□ MVAC therapy<br>□ Other<br>( )				□ CR<br>□ PR<br>□ SD<br>□ PD<br>□ Unknown	□ CR<br>□ PR<br>□ SD<br>□ PD<br>□ Unknown	□ Completion<br>□ Intolerance<br>□ Other<br>( )<br>□ Unknown

*: Dose and number of cycles (if chemotherapy)

Surgical therapy for the target disease

#	Resection site	Date (dd/MMM/yyyy)
1	□ Bladder<br>□ Lymph node<br>□ Other ( )	
2	□ Bladder<br>□ Lymph node<br>□ Other ( )	
3	□ Bladder<br>□ Lymph node<br>□ Other ( )	
4	□ Bladder<br>□ Lymph node<br>□ Other ( )	
5	□ Bladder<br>□ Lymph node<br>□ Other ( )	

	Name (print)	Signature	Date form completed or verified (dd/MMM/yyyy)
Investigator			
Subinvestigator			
Study coordinator			

Appendix 7 Country-Specific Information

Japan

The amendment 1 includes the following country-specific information in order to follow an advice from the Pharmaceuticals and Medical Devices Agency (PMDA, Japanese regulatory authority) at the initial clinical trial notification filing:

Among contraception as described in Section 4.3, contraception approved in Japan are the following: barrier contraceptives (male condom or diaphragm); hormonal contraceptives (combination oral contraceptives); or intrauterine devices.

Appendix 8 Sponsor Signature

Approval of the Protocol

Product Name: S-588410
Study Protocol Title: A multicenter, open-label, phase 2 study of S-588410 as maintenance monotherapy after first-line platinum-containing chemotherapy in patients with advanced and/or metastatic bladder cancer
Study Protocol Number: 1408P1921
Version Number: Amendment 6
Issue Date of Original: 25 December 2013
Issue Date of Latest Amendment: 1 April 2017

Sponsor signatory:

This clinical study protocol was subject to critical review and has been approved by the sponsor:

Refer to electronic signature page
[redacted]
Chief Medical Officer
Shionogi & Co. Ltd.
[redacted]

Refer to electronic signature page
Date: day-month-year

Signature Page

Document Information

Short Title :	Clinical Study Protocol: 1408P1921, Phase 2-Amendment 7
Name :	E_PC_P1921_Ph2 Version 8 (Amendment 7)
Object ID :	0900aabf80d6e16b

Signature

Date : (GMT) Apr 03, 2017 04:06:17

Signed by : (Name and ID) [redacted]

Justification : Approved as Chief Medical Officer

Electronic signature page generated by MIND
