# Supplementary material for: A Phase 2 Study of S-588410 Maintenance Monotherapy for Platinum-Treated Advanced or Metastatic Urothelial Carcinoma
Source: Bladder Cancer. 2022 Jun 3;8(2):179–92. doi: 10.3233/BLC-211592 (PMC11181746; doi:10.3233/BLC-211592)
Supplement: Supplementary Tables [file blc-8-blc211592-s002.docx]

Table S1. TNM classification at enrollment for all participants

| TNM_T | TNM_N | TNM_M | S-588410  (n=45) | Observation  (n=36) | Total  (n=81) |
| --- | --- | --- | --- | --- | --- |
| Tx | N0 | M0 | 8 (17.8) | 5 (13.9) | 13 (16.0) |
| T0 | N0 | M0 | 5 (11.1) | 2 (5.6) | 7 (8.6) |
| T2 | N0 | M0 | 0 | 1 (2.8) | 1 (1.2) |
| T3 | N0 | M0 | 1 (2.2) | 1 (2.8) | 2 (2.5) |
| T4 | N0 | M0 | 1 (2.2) | 2 (5.6) | 3 (3.7) |
| Tx | N1 | M0 | 1 (2.2) | 2 (5.6) | 3 (3.7) |
| Tx | N2 | M0 | 3 (6.7) | 2 (5.6) | 5 (6.2) |
| T0 | N1 | M0 | 0 | 1 (2.8) | 1 (1.2) |
| T2 | N2 | M0 | 0 | 1 (2.8) | 1 (1.2) |
| T3 | N1 | M0 | 1 (2.2) | 0 | 1 (1.2) |
| T3 | N2 | Mx | 0 | 1 (2.8) | 1 (1.2) |
| Tx | N0-3 | M1 | 13 (28.9) | 9 (25.0) | 22 (27.2) |
| T0 | N0-3 | M1 | 5 (11.1) | 4 (11.1) | 9 (11.1) |
| T1-3 | N0-3 | M1 | 7 (15.6) | 5 (13.9) | 12 (14.8) |

Data are n (%)

Table S2 Anti-tumor response assessed by RECIST version 1.1

|  | **S-588410 (n=45)** | **Observation (n=36)** |
| --- | --- | --- |
| Best overall response |  |  |
| CR | 1 (2.2) | 0 |
| PR | 3 (6.7) | 0 |
| SD | 6 (13.3) | 5 (13.9) |
| Non-CR/Non-PD | 1 (2.2) | 0 |
| PD | 23 (51.1) | 14 (38.9) |
| No disease | 9 (20.0) | 13 (36.1) |
| Not evaluable | 2 (4.4) | 4 (11.1) |
| Response rate (CR+PR) | 4 (8.9) | 0 |
| 90% CI | (3.1–19.2) | (0.0–8.0) |
| Disease control rate (CR+PR+SD+Non-CR/Non-PD) | 11 (24.4) | 5 (13.9) |
| 90% CI | (14.4–37.2) | (5.6–27.0) |

Data are n (%) unless otherwise stated. Abbreviations: CI = confidence interval, CR = complete response; PD = progressive disease; PR = partial response; RECIST = response evaluation criteria in solid tumors; SD, stable disease.

Table S3. TNM classification at enrollment and tumor response for the 12 participants who had completed the study

| No. | Group | TNM_T | TNM_N | TNM_M | BOR |
| --- | --- | --- | --- | --- | --- |
| 1 | S-588410 | Tx | N0 | M0 | ND |
| 2 | S-588410 | T0 | N0 | M0 | ND |
| 3 | S-588410 | T0 | N0 | M0 | irPD* |
| 4 | S-588410 | Tx | N0 | M1 | ND |
| 5 | S-588410 | Tx | N0 | M1 | irPR |
| 6 | S-588410 | T0 | N0 | M1 | irPR |
| 7 | S-588410 | T0 | N0 | M1 | irSD |
| 8 | S-588410 | T2 | N0 | M1 | irSD |
| 9 | Observation | Tx | N0 | M0 | ND |
| 10 | Observation | T0 | N0 | M0 | ND |
| 11 | Observation | T0 | N0 | M0 | ND |
| 12 | Observation | Tx | N1 | M0 | ND |

*ND until a new lung lesion was detected at week 96. Abbreviations: BOR = best overall response; irPD = immune-related progressive disease; irPR = immune-related partial response; irSD = immune-related stable disease; ND = no disease.
